# Deficiency of CAMSAP2 impairs olfaction and the morphogenesis of mitral cells

**DOI:** 10.1038/s44319-024-00166-x

**Published:** 2024-06-05

**Authors:** Zhengrong Zhou, Xiaojuan Yang, Aihua Mao, Honglin Xu, Chunnuan Lin, Mengge Yang, Weichang Hu, Jinhui Shao, Peipei Xu, Yuejia Li, Wenguang Li, Ruifan Lin, Rui Zhang, Qi Xie, Zhiheng Xu, Wenxiang Meng

**Affiliations:** 1 State Key Laboratory of Molecular Developmental Biology, Institute of Genetics and Developmental Biology, Chinese Academy of Sciences, 100101 Beijing, China; 2 Neuroscience Center, Department of Basic Medical Sciences, Shantou University Medical College, 515041 Shantou, Guangdong China; 3 University of Chinese Academy of Sciences, 100049 Beijing, China; 4 Biology Department, College of Sciences, Shantou University, 515063 Shantou, China; 5 Animal Laboratory Center, Institute of Genetics and Developmental Biology, Chinese Academy of Sciences, 100101 Beijing, China; 6 Chinese Academy of Chinese Medical Sciences, 100700 Beijing, China; 7 Innovation Academy for Seed Design, Chinese Academy of Sciences, 100101 Beijing, China

**Keywords:** CAMSAP2, Mitral Cell, Odor-dependent Behaviors, Morphogenesis, Cell Adhesion, Polarity & Cytoskeleton, Development, Neuroscience

## Abstract

In developing olfactory bulb (OB), mitral cells (MCs) remodel their dendrites to establish the precise olfactory circuit, and these circuits are critical for individuals to sense odors and elicit behaviors for survival. However, how microtubules (MTs) participate in the process of dendritic remodeling remains elusive. Here, we reveal that calmodulin-regulated spectrin-associated proteins (CAMSAPs), a family of proteins that bind to the minus-end of the noncentrosomal MTs, play a crucial part in the development of MC dendrites. We observed that *Camsap2* knockout (KO) males are infertile while the reproductive tract is normal. Further study showed that the infertility was due to the severe defects of mating behavior in male mice. Besides, mice with loss-of-function displayed defects in the sense of smell. Furthermore, we found that the deficiency of CAMSAP2 impairs the classical morphology of MCs, and the CAMSAP2-dependent dendritic remodeling process is responsible for this defect. Thus, our findings demonstrate that CAMSAP2 plays a vital role in regulating the development of MCs.

## Synopsis

CAMSAP2, the non-centrosomal microtubule binding protein, has critical roles in the development of mitral cells, establishment of the olfactory circuitry and olfactory-guided mating behavior.

- CAMSAP2 knockout male mice exhibit infertility.
- Deficiency in CAMSAP2 impairs olfaction.
- Deficiency in CAMSAP2 impairs olfactory-guided mating behavior of male mice.
- CAMSAP2 plays a critical role in the development of mitral cells in the olfactory bulb.

## Abstract (web summary)

CAMSAP2, the non-centrosomal microtubule binding protein, has critical roles in the development of mitral cells, establishment of the olfactory circuitry and olfactory-guided mating behavior.

## Graphical abstract

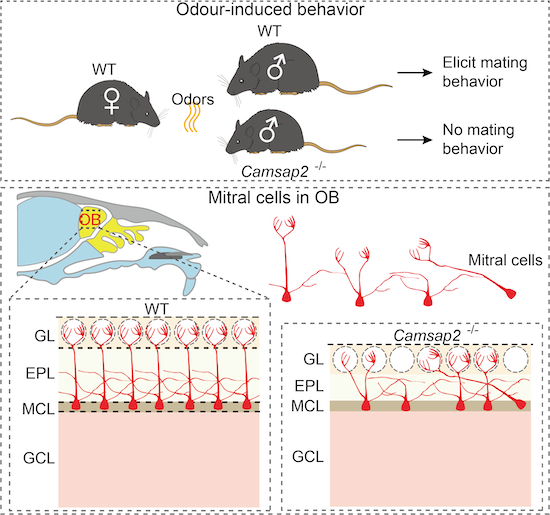

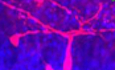

## Introduction

In the mammalian olfactory system, the odorant receptors (ORs) in the nasal cavity recognize chemical cues and relay the information to the OB. After processing in the OB, mitral cells and tufted cells (MTCs), the only two projection neurons in OB, convey the odor information to the olfactory cortex to induce distinct behaviors (Chen and Hong, 2018; Dulac and Wagner, 2006). Olfactory sensory neurons (OSNs) expressing the same OR converge and send their axons to two specific sites of the OB to form a glomerular structure (Mombaerts et al, 1996). In the OB, MTCs extend a single apical dendrite radially into the glomerulus to receive the odor information (Dulac and Wagner, 2006; Imai, 2014). As OSN axons in one glomerulus represent one OR, and each MTC projects its primary dendrite to only one glomerulus, this junction constructs the precise connection between ORs and olfactory cortex (Imamura et al, 2011). However, how neurons shape the precise connection between OSNs and MTCs remains elusive.

During embryonic development, the MCs migrate from the olfactory ventricle to the mitral cell layer (MCL), and extend dendrites into multiple glomeruli (Blanchart et al, 2006; Hinds, 1968). After the process of pruning in the first postnatal week, these projection neurons form the typical mature morphology to connect with OSNs and interact with local interneurons to construct olfactory circuits (Imai, 2014; Lledo et al, 2005). The odorous signal input from the glomerulus is first processed by local circuits before it is relayed by projection neurons to the olfactory cortex. Thus, the precise morphogenesis of projection neurons, that is, one apical dendrite extended into the glomerulus and several lateral dendrites extended into the external plexiform layer (EPL), is critical for odor information processing and subsequent behaviors. Abolishment of odorant-evoked activity only delays but does not perturb the pruning of MCs (Lin et al, 2000; Matsutani and Yamamoto, 2000), and genetic manipulation alters the organization of MCs (Inokuchi et al, 2017; Kobayakawa et al, 2007). Moreover, the BMP and Notch pathways were reported to regulate the morphogenesis of MCs dendrites (Aihara et al, 2021; Muroyama et al, 2016). Besides, activity-dependent local protection participates in this remodeling process, mainly via regulating the formation of F-actin (Aihara et al, 2021; Fujimoto et al, 2023). However, whether and how MTs regulate the remodeling of MCs remains unclear.

The CAMSAPs are MT minus-ends binding proteins and play a vital role in regulating the MT network (Akhmanova and Hoogenraad, 2015; Meng et al, 2008; Tanaka et al, 2012; Zhou et al, 2020). Unlike *Drosophila* and *C. elegans*, mammals have three homologs, CAMSAP1, CAMSAP2 (CAMSAP1L1) and CAMSAP3 (Nezha) (Akhmanova and Hoogenraad, 2015). Though the structural domains of CAMSAP proteins are similar, they are non-redundant proteins as a series of discrepancies exist among them. First, their behavior is different. CAMSAP1 only tracks the minus-ends of MTs while CAMSAP2 and CAMSAP3 decorate and stabilize the minus-ends (Hendershott and Vale, 2014; Jiang et al, 2014; Tanaka et al, 2012); Second, they have different expressions, locations and distributions in cells and tissue (Ho et al, 2023; Pongrakhananon et al, 2018; Wu et al, 2016; Zhou et al, 2020). Finally, their functions are divergent. CAMSAP1 regulates neuronal migration and polarization, while CAMSAP3 is only responsible for neuronal polarization (Khalaf-Nazzal et al, 2022; Pongrakhananon et al, 2018; Zhou et al, 2020). Besides, the previous study and our recent work also show that CAMSAP1 but not CAMSAP2 and CAMSAP3 is responsible for spermatogenesis (Hu et al, 2023; Robinson et al, 2020). These features suggest that CAMSAPs are non-redundant in mammals and regulate mammals’ development in a sophisticated manner. Previous studies showed that the deficiency of CAMSAP1 results in epileptic seizure and neuronal migration disorder (Khalaf-Nazzal et al, 2022; Zhou et al, 2020), while *Camsap3* knockout (KO) mice exhibit primary ciliary dyskinesia (Robinson et al, 2020). In this study, we set out to similarly address the function of CAMSAP2 and its potential role in organismal development.

Herein described, we construct *Camsap2* KO and conditional knockout (cKO) mice to investigate the function of CAMSAP2 in organismal development. We observed that a deficiency of CAMSAP2 abolished the mating behavior of male mice and led to infertility. *Camsap2* deletion perturbs the morphogenesis of MC dendrites and the sense of smell.

## Results and discussion

### *Camsap2* KO males are infertile, while spermatogenesis is normal

**Figure 1:**
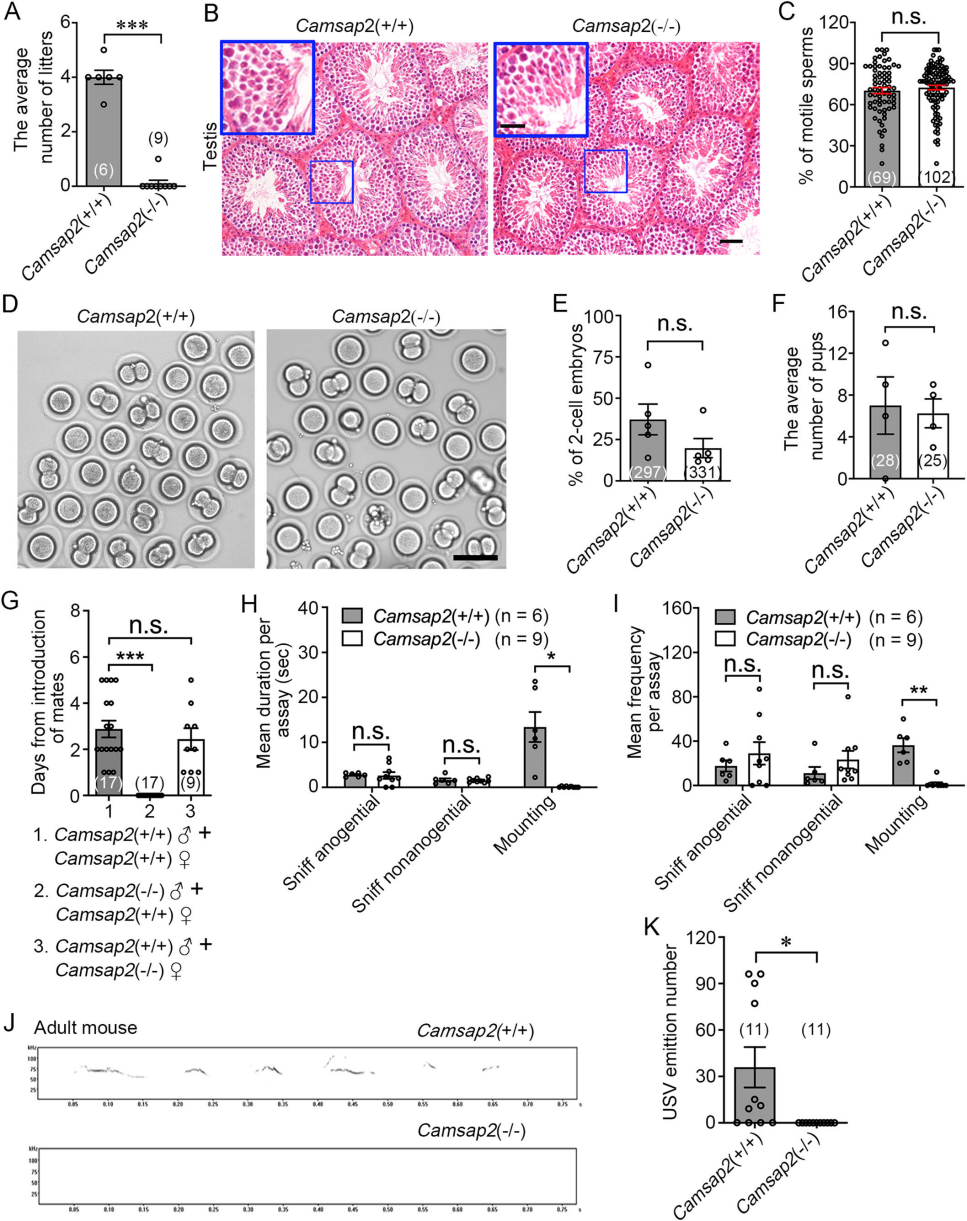
Copulatory behavior but not spermatogenesis is responsible for the infertility of *Camsap2* KO males. (**A**) Quantification of the average number of litters produced by male mice (unpaired Student’s *t* test, *n* = 6 or 9 biological replicates, ***P = 2.84 × 10^-6^). (**B**) Representative images of testicular histology sections stained with H&E staining. (**C**) The percentage of motile sperms produced by *Camsap2*(+/+) and *Camsap2*(−/−) mice (unpaired Student’s *t* test, *n* = 4 biological replicates). (**D**) Representative images of 2-cell embryos produced by IVF. (**E**) The percentage of 2-cell embryos produced by IVF (unpaired Student’s *t* test, *n* = 5 biological replicates). (**F**) The average number of pups produced by IVF (unpaired Student’s *t* test, *n* = 4 biological replicates). (**G**) Mice mating assay. Average number of days after pairing until the copulatory plug appears, 0. indicates that no plugs appeared after a maximum time span of 5 days of monitoring (one-way ANOVA with Dunnett’s multiple comparisons test, *n* = 9–17 biological replicates, ***P < 0.0001). (**H**, **I**) The duration (**H**) and frequency (**I**) of mating subroutines in WT and KO mice (unpaired Student’s *t* test, *n* = 6 or 9 biological replicates, *P = 0.0102, **P = 0.00233). (**J**) Representative spectrograms of USVs emitted by adult male mice. (**K**) Quantification of the number of USVs emitted by adult male mice (unpaired Student’s *t* test, *n* = 11 biological replicates, *P = 0.0208). Data information: Data are represented as mean ± SEM. n.s. not significant, *P* > 0.05. Scale bars: (**B**) 100 μm and 50 μm in full-size images and zoomed areas; (**D**) 50 μm. Source data are available online for this figure.

To investigate the function of CAMSAP2, we generated a line of *Camsap2* null allele mice via CRISPR/Cas9 (Appendix Fig. S1A). Animals carrying both null alleles are designated *Camsap2*(−/−) (or *Camsap2* KO) (Appendix Fig. S1A–C). In comparison with the slight body weight reduction in *Camsap2*(−/−), the most significant phenotype is the sterility of *Camsap2*(−/−) males, as we could not obtain pups with those males (Fig. 1A; Appendix Fig. S1D,E).

Considering CAMSAP2 is a noncentrosomal MT minus-ends binding protein, and the spermatozoa contain abundant MT structures, such as the manchette and the sperm flagella (Lehti and Sironen, 2016; O’Donnell, 2014; Rosenbaum and Witman, 2002), the most reasonable cause of male sterility in *Camsap2*(−/−) mice was the impairment of sperm formation. We therefore performed Hematoxylin and eosin (H&E) staining to analyze the structure of the testis and sperm, but found that both were normal in *Camsap2*(−/−) males (Fig. 1B), and the percentage of motile sperm from *Camsap2*(−/−) males were comparable to wildtype (WT) (Fig. 1C). To further confirm that the sperm from *Camsap2*(−/−) males was fully functional, we performed in vitro fertilization (IVF) assays and found that the percentage of 2-cell embryos was similar to the WT condition (Fig. 1D,E). Furthermore, sperm from *Camsap2*(+/+) or *Camsap2*(−/−) used in artificial insemination led to roughly equal numbers of successfully born pups (Fig. 1F). Collectively, these results indicate that CAMSAP2 controls male reproduction independent of the reproductive tract.

### Ablation of CAMSAP2 alters the sexual behavior of male mice

Since the sperm from *Camsap2*(−/−) males can generate offspring via the artificial insemination, we suspected that the deficiency of CAMSAP2 may change the copulatory behavior of male mice. To test this assumption, male mice were paired with female mice and the presence of vaginal plugs (indicative of ejaculation) was assessed for consecutive 5 days. The production of plugs caused by *Camsap2*(+/+) males was detected at a mean of 3 days (Fig. 1G). However, we could not detect vaginal plugs in females paired with *Camsap2*(−/−) males (Fig. 1G), indicating that *Camsap2*(−/−) males did not successfully mate with females. In male mice, copulation involves approach and investigation, emission of ultrasonic vocalizations (USVs), mounting, intromission and ejaculation (Chen and Hong, 2018; Hull and Dominguez, 2007). To find the defective phase, we then analyze the sexual behavior of males with video recordings. Both *Camsap2*(+/+) and *Camsap2*(−/−) males showed no difference in duration and frequency of sniffing, while the mounting behavior was almost completely absent in *Camsap2*(−/−) males (Fig. 1H,I). Furthermore, we examined the USVs emitted by male mice in response to female mice, which is regarded as a male-typical precopulatory reproductive behavior normally accompanied by sexual chasing, preceding mounts or intromission. Besides, male who emits USVs often exhibit higher attractiveness to female and this process also enhances sexual receptivity in female (Asaba et al, 2014; Nyby et al, 1992; Pomerantz et al, 1983). During the experiment, most *Camsap2*(+/+) males vocalized intensively, whereas none of the *Camsap2*(−/−) males vocalized (Fig. 1J,K). To exclude the possibility that loss of CAMSAP2 destroys the ability to generally produce ultrasound, P6 mice pups were separated from their mother and both *Camsap2*(+/+) and *Camsap2*(−/−) mice emitted ultrasound as a separation response (Fig. EV1A,B). These results indicate the deficiency of CAMSAP2 impairs the activation of sexual motivation in male mice.

**Figure EV1:**

Hormone levels and motor function are not responsible for the infertility of *Camsap2*(−/−) male mice. (**A**) Representative spectrograms of USVs emitted by P6 mice. (**B**) Quantification of the number of USVs emitted by P6 mice (unpaired Student’s *t* test, *n* = 5 or 12 biological replicates). (**C**) The concentration of serum testosterone (unpaired Student’s *t* test, *n* = 18 or 25 biological replicates). (**D**) The concentration of serum estradiol (unpaired Student’s *t* test, *n* = 11 or 13 biological replicates). (**E**) Motor performance on an accelerating rotarod, no significant difference exists at any time point (two-way R-M ANOVA, *n* = 9 biological replicates). Data information: Data are represented as mean ± SEM. n.s. not significant, *P* > 0.05.

Steroid hormones activate the emission of USV and mounting in response to stimulus females. Moreover, the gonadal steroid hormones also play a critical role in the development and function of these behavior-associated neural circuits (Hull and Dominguez, 2007; Nyby et al, 1992; Wu et al, 2009), we then assayed the titers of hormones in serum. Unexpectedly, the titers of circulating testosterone and estradiol in *Camsap2*(−/−) males were comparable to those of *Camsap2*(+/+) males (Fig. EV1C,D). As motor deficit or muscular weakness may limit the mounting behavior, we also assayed their motor performance on the rotarod, and *Camsap2*(−/−) males performed equivalently to *Camsap2*(+/+) males (Fig. EV1E). Taken together, these results suggest that CAMSAP2 is required for sexual behaviors.

### Loss of CAMSAP2 impairs the sense of smell

**Figure 2:**
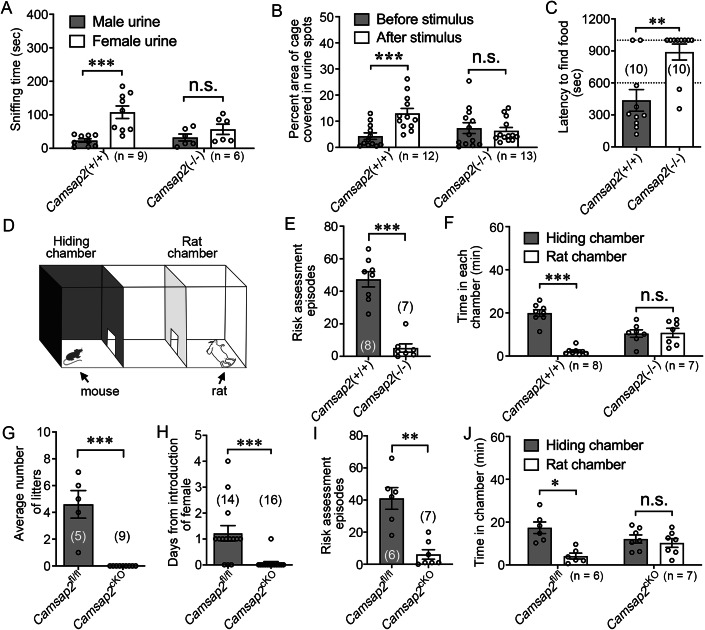
Deficiency of CAMSAP2 dysregulated olfactory behaviors. (**A**) Total amount of time that male mice spend sniffing male or female urine (ratio paired Student’s *t* test, *n* = 9 or 6 biological replicates, ***P = 0.0001). (**B**) Territorial marking assay. Percent area of cage covered by male urine in response to a spot of female urine (ratio paired Student’s *t* test, *n* = 12 or 13 biological replicates, ***P = 0.0006). (**C**) Buried food test. The latency for mice to locate the food pellet. The dotted line at 600 indicates the end time of the experiment, and the dotted line at 1000 indicates the mice do not find the hidden food within the allotted time (unpaired Student’s *t* test, *n* = 10 biological replicates, **P = 0.00233). (**D**) Schematic diagram of the defensive behavior test arena. The mouse and rat in each chamber are indicated by arrows. (**E**) Quantification of risk assessment behavior (unpaired Student’s *t* test, *n* = 8 or 7 biological replicates, ***P = 8.95 × 10^−6^). (**F**) Quantification of the time spent by mice in the hiding chamber and rat chamber (ratio paired Student’s *t* test, *n* = 8 or 7 biological replicates, ***P = 0.0001). (**G**) Quantification of the average number of litters produced by *Camsap2*^fl/fl^ and *Camsap2*^cKO^ male mice, 0 indicate that no plugs appeared after a maximum time span of 6 days of monitoring (unpaired Student’s *t* test, *n* = 5 or 9 biological replicates, ***P = 0.0005). (**H**) Mice mating assay of *Camsap2*^fl/fl^ and *Camsap2*^cKO^ mice. The average number of days after pairing until the copulatory plug appears with a maximum time span of 6 days (unpaired Student’s *t* test, *n* = 14 or 16 biological replicates, ***P < 0.0001). (**I**) Quantification of risk assessment behavior of *Camsap2*^fl/fl^ and *Camsap2*^cKO^ mice (unpaired Student’s *t* test, *n* = 6 or 7 biological replicates, **P = 0.00226). (**J**) Quantification of the time spent by *Camsap2*^fl/fl^ and *Camsap2*^cKO^ male mice in the hiding chamber and rat chamber (ratio paired Student’s *t* test, *n* = 6 or 7 biological replicates, *P = 0.026). Data information: Data are represented as mean ± SEM. n.s. not significant, *P* > 0.05. Source data are available online for this figure.

As *Camsap2*(−/−) males sniff but do not mate with the receptive females (Fig. 1G–I), we then asked whether it is an olfaction defect that might cause these results. To test this hypothesis, *Camsap2*(+/+) males and *Camsap2*(−/−) males were placed in a cage that contained male and female urine. *Camsap2*(+/+) males discriminate between the two types of urine and show a higher preference for female urine, while *Camsap2*(−/−) males cannot distinguish between them (Fig. 2A). Subsequently, we examined the territorial marking behavior, a male-typical territorial behavior of depositing urine spots in response to the estrous female scent (Schoeller et al, 2016). We found that *Camsap2*(+/+) males robustly marked their territory, while *Camsap2*(−/−) males did not enhance their marking behavior when female urine was introduced into the cage (Fig. 2B).

As most mammalian species have two olfactory circuits, the accessory system to sense the non-volatile pheromonal odorants from conspecifics and the main olfactory system to detect non-pheromonal odors from food and predators which is relevant to species survival (Baum and Cherry, 2015), we then asked whether both olfactory circuits are impaired in *Camsap2*(−/−) mice. To answer this question, a buried food test was performed to test general olfactory function, and the latency for finding the hidden food was significantly longer in *Camsap2*(−/−) mice (Fig. 2C). Defensive behaviors, including avoidance and risk assessment, are elicited when mice sensed their predators’ odors (Papes et al, 2010; Schoeller et al, 2016). We, therefore, placed individual mice in a three-chamber cage with a live (anesthetized) rat, which is considered a natural mouse predator. *Camsap2*(+/+) mice displayed significant innate risk assessment and avoidance behaviors and spent most of the test time in the hiding chamber. In contrast, the *Camsap2*(−/−) mice did not display these defensive behaviors, rather, they directly investigated the rat without apparent caution or retreating (Fig. 2D–F). Together, our results demonstrate that deficiency of CAMSAP2 causes a general olfactory defect, which leads to *Camsap2*(−/−) mice being unable to detect the odors of conspecifics, predators and food.

To confirm that the abnormal behaviors mentioned above are due to the defects of the nerve system, we generated a conditional allele with LoxP sites flanking *Camsap2* exon 2 (Appendix Fig. S2A,B). These mice were crossed with the Nesin-Cre line, a pan-neural line that expresses Cre-recombinase throughout the brain, and bred to obtain homozygous *Camsap2*^fl/fl^; Nestin-Cre (denoted *Camsap2*^cKO^) mice (Appendix Fig. S2C). *Camsap2*^cKO^ mice show smaller brain and body sizes compared to *Camsap2*^fl/fl^ mice (Appendix Fig. S2D-F). In correspondence with *Camsap2* null allele mice, *Camsap2*^cKO^ mice are also infertile (Fig. 2G), and almost none of them produce vaginal plugs after cohabiting with female mice (Fig. 2H). In addition, *Camsap2*^cKO^ mice did not display innate risk assessment and avoidance behaviors (Fig. 2I,J). Thus, by analyzing the behaviors of mice, we have determined that the expression of CAMSAP2 in the nerve system is required for the sense of smell.

### CAMSAP2 is required for the development of MCs

As the OB is an important station for odor information processing and delivery. We were then curious whether the loss of CAMSAP2 affects the OB. As we expected, the structure of the OB was changed (Fig. EV2A,B), and especially the thickness of the EPL was significantly reduced in knockout mice (Fig. EV2A–E).

**Figure EV2:**

Knockout of *Camsap2* diminishes the thickness of EPL in the OB. (**A**) Schematic diagram of the OB. (**B**) Representative images of the OB from *Camsap2*^fl/fl^ and *Camsap2*^cKO^ mice, mature neurons were immunostained with antibody anti-NeuN. (**C**) Quantification of the thickness of EPL from *Camsap2*^fl/fl^ and *Camsap2*^cKO^ mice (two-way R-M ANOVA with Šídák’s multiple comparisons test, *n* = 3 biological replicates, ***P = 0.0009 or < 0.0001, **P = 0.0033). (**D**) Quantification of the area of the OB from *Camsap2*^fl/fl^ and *Camsap2*^cKO^ mice (unpaired Student’s *t* test, *n* = 3 biological replicates, *P = 0.0221). (**E**) Quantification of the area surrounded by MCL from *Camsap2*^fl/fl^ and *Camsap2*^cKO^ mice (unpaired. Student’s *t* test, *n* = 3 biological replicates). Data information: Data are represented as mean ± SEM. n.s. not significant, *P* > 0.05. Scale bars: 400 μm and 100 μm in full-size images and zoomed areas.

**Figure 3:**
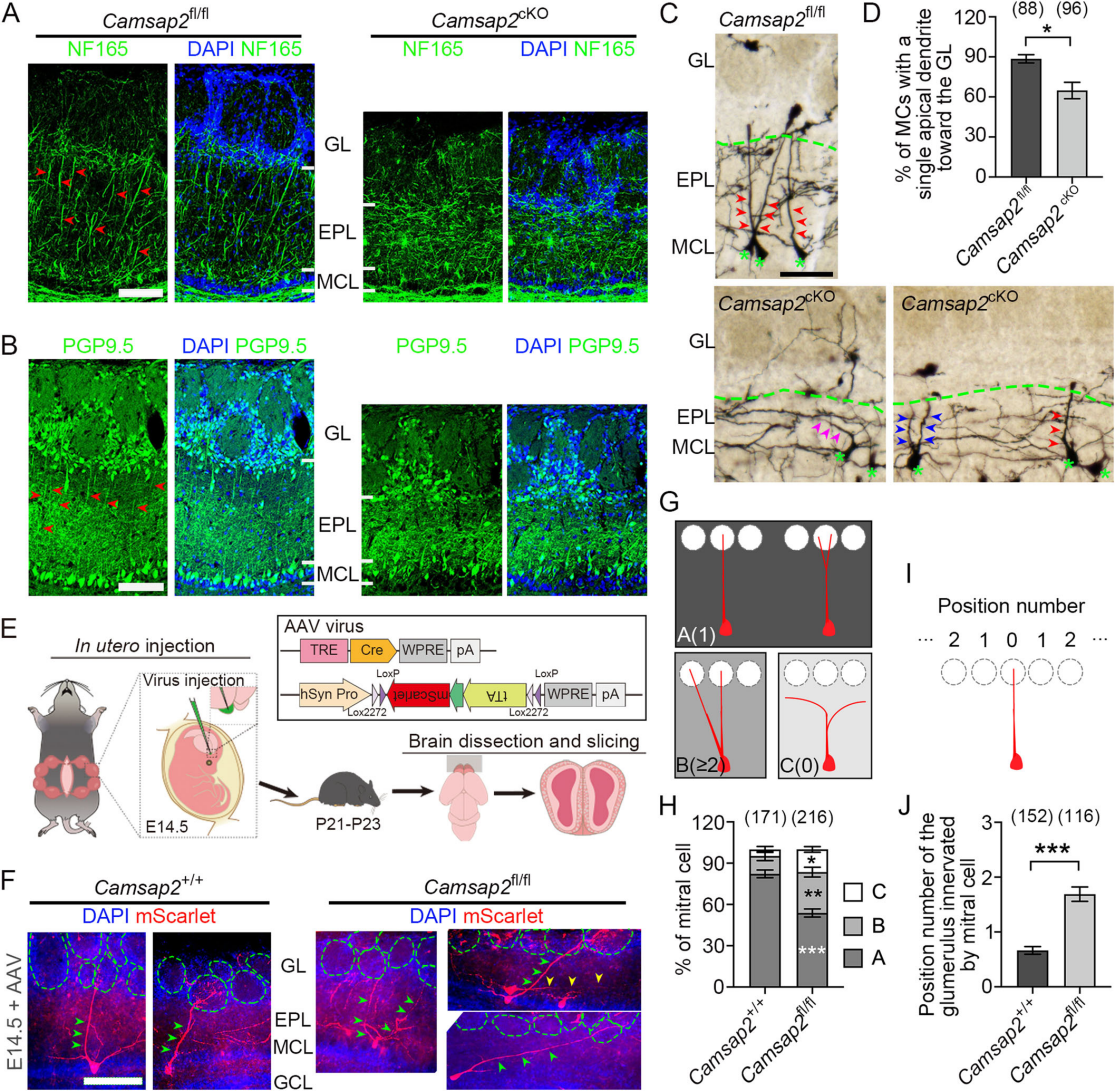
CAMSAP2 deficiency disturbs the apical dendritic projection of MCs. (**A**, **B**) Representative images of immunofluorescence staining for NF165 (**A**) and PGP9.5 (**B**) in OB coronal sections. Red arrowheads indicate the perpendicular apical dendrites of MCs. (**C**) Neurons in the OB were stained by Golgi-cox staining. Green asterisks indicate the MCs, red arrowheads indicate the single apical dendrites extending from the MC straight toward the GL, blue arrowheads indicate two apical dendrites extending from the single MC, pink arrowheads indicate single apical dendrites extending towards the lateral side of EPL. Green dashed lines indicate the boundary of GL/EPL. (**D**) Quantification of the percentage of MCs with a perpendicular single apical dendrite (unpaired Student’s *t* test, *n* = 5 biological replicates, *P = 0.0137). (**E)** Schema showing AAV-based sparse labeling. An AAV mixture was injected into the olfactory ventricle at E14.5, the neuronal morphology was analyzed at P21-P23. (**F**) Representative images of MCs labeled by mScarlet. Apical dendrites extending toward GL are indicated by green arrowheads, and an apical dendrite extending toward the lateral side is indicated by yellow arrowheads. Glomeruli are depicted by green dashed circles. (**G**) Apical dendrite morphologies of MCs were divided into 3 types: Type A, apical dendrite projecting into a single glomerulus; Type B, apical dendrite projecting into multiple glomeruli; Type C, apical dendrite does not project into any glomerulus. (**H**) Quantification of the percentage of MCs with different types of apical dendrite (two-way ANOVA with Šídák’s multiple comparisons test, *n* = 6 or 5 biological replicates, *P = 0.0228, **P = 0.001, ***P < 0.0001, detailed information is reported in Source data). (**I**) Schema showing the position number of glomeruli innervated by an MC. (**J**) Quantification of the position number of glomeruli innervated by MCs (unpaired Student’s *t* test, *n* = 6 or 5 biological replicates, ***P = 7.75 × 10^−11^). Data information: Data are represented as mean ± SEM. Scale bars, 100 μm. Source data are available online for this figure.

EPL is a neuropil layer that mainly contains the dendrites and synapses of MTC, MTC are the only two projection neurons in the OB that directly receive excitatory input from OSNs. After processing, they convey sensory information to the olfactory cortex (as MCs and TCs are similar in morphology and function, hereafter, we focus our research only on MCs). We then investigated whether the *Camsap2* gene disruption in MCs leads to variation in the anatomical properties of MCs. We first immunostained with anti-neurofilament 165 (NF165) to label the dendrites in the EPL. In control mice, MCs sent thick apical dendrites straight toward the GL (Fig. 3A). In contrast, the classical characteristic of apical dendrites disappeared in *Camsap2*^cKO^ mice (Fig. 3A). Subsequently, we analyzed MCs and their dendrites by staining with anti-PGP9.5, a specific marker for MCs (Taniguchi et al, 1993). The apical dendrites were easily visualized in control mice, while they became indistinct in *Camsap2*^cKO^ mice (Fig. 3B).

To investigate the morphological changes of single MCs, we stained the OB by Golgi-cox staining. The typical mature morphology of MCs was seen in *Camsap2*^fl/fl^ mice. However, the proportion of MCs with normal morphology in *Camsap2*^cKO^ was lower than that in *Camsap2*^fl/fl^ (Fig. 3C,D), and we found some MCs in *Camsap2*^cKO^ showing atypical morphology, such as multiple apical dendrites or the apical dendrite extending toward the lateral side (Fig. 3C). As Golgi-cox staining sporadically labels all neurons and their processes in the OB, this feature impeded us from acquiring comprehensive information about single MCs. To resolve this problem, we combined the AAV-mediated gene delivery system and the supernova system to specifically label MCs at single-cell resolution (Luo et al, 2016; Togashi et al, 2020). The virus mixture was unilaterally injected to the OB ventricle at E14.5, and the OB was collected at postnatal day (P) 21–23. After immunostaining and a tetrahydrofuran-based clearing procedure (Erturk et al, 2011), the morphology of MCs was analyzed (Fig. 3E). Corresponding to the results of Golgi-cox staining, mScarlet labeled MCs showed multiple morphologies in MC-specific knockout mice (Fig. 3F), which we then categorized into three types (Fig. 3G). Compared with the MCs in WT mice, MCs in MC-specific knockout mice showed a higher proportion of type B and C (Fig. 3H). Furthermore, we also found that many more MCs in MC-specific knockout mice tend to project to a neighboring glomerulus (Fig. 3F,I,J), and select adjacent targets requiring longer dendrites (Position number 1 or 2, Fig. 3I). Even though we do not analyze the lateral dendrites of MCs, the narrowed EPL suggests that deficiency of CAMSAP2 impairs the development of lateral dendrites. Collectively, our data show that CAMSAP2 participates in the morphogenesis of MCs.

### CAMSAP2 deficiency alters dendritic remodeling

**Figure 4:**
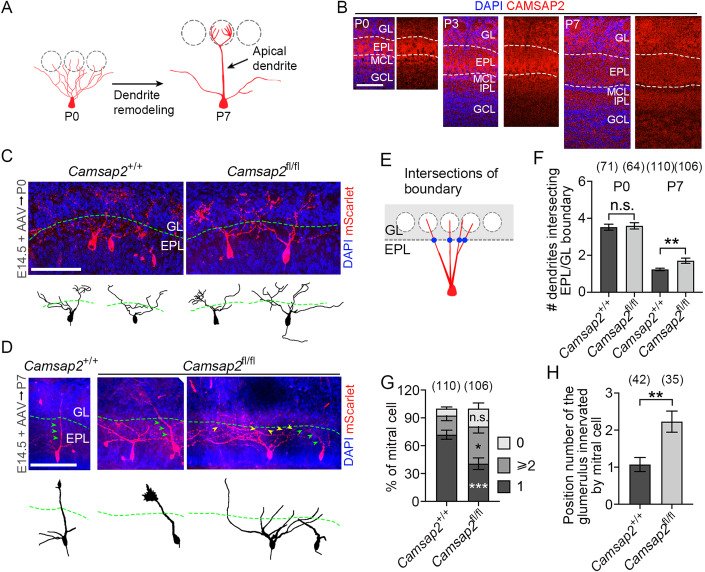
CAMSAP2 deficiency disrupts dendritic remodeling. (**A**) Development of MC from P0 to P7. At P0, the MC develops widespread apical dendrites into multiple glomeruli, at P7, the MC forms typical mature morphology with a single apical dendrite and several lateral dendrites via dendritic remodeling. (**B**) Distribution of CAMSAP2 in coronal sections of the OB at P0, P3, and P7. (**C**, **D**) Representative images of MCs labeled by mScarlet. Green arrowheads indicate single apical dendrites extending from the MC toward the GL, and yellow arrowheads indicate multiple apical dendrites from single MC extending toward the GL. Green dashed lines indicate the boundary of GL/EPL. Representative MCs are reconstructed and shown below. (**E**) Intersections between dendrites and the GL/EPL boundary are shown as a cartoon. (**F**) Quantification of the number of crossing sites at the GL/EPL border in P0 and P7 mice (two-way ANOVA with Šídák’s multiple comparisons test, P0, *n* = 4 or 6 biological replicates; P7, *n* = 7 or 6 biological replicates, **P = 0.0098, detailed information is reported in Source data). (**G**) Quantification of the percentage of MC with different number of intersections in P7 mice (two-way ANOVA with Šídák’s multiple comparisons test, *n* = 7 or 6 biological replicates, *P = 0.0444, ***P = 0.0007, detailed information is reported in Source data). (**H**) Quantification of the position number of glomeruli innervated by MC apical dendrites (unpaired Student’s *t* test, *n* = 7 or 6 biological replicates, **P = 0.00127). Data information: Data are represented as mean ± SEM. n.s. not significant, *P* > 0.05. Scale bars, 100 μm. Source data are available online for this figure.

**Figure EV3:**
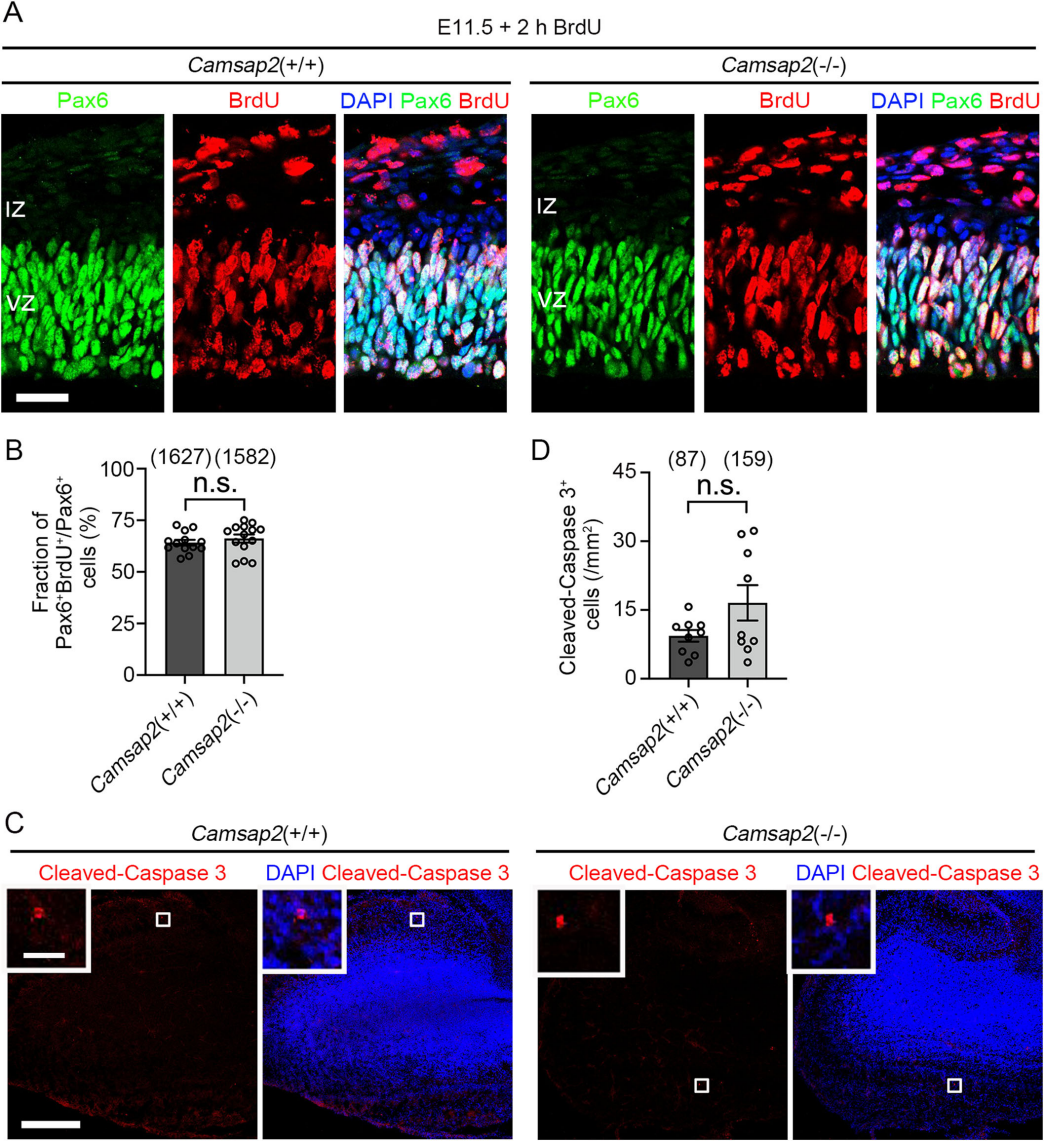
CAMSAP2 is dispensable for the neurogenesis and apoptosis of MCs. (**A**) Representative images of coronal OB sections from E11.5 mice. The progenitor cells and proliferating. cells are immunostained with antibodies against Pax6 and BrdU after BrdU labeling for 2 h. (**B**) The percentage of proliferating progenitor cells (unpaired Student’s *t* test, *n* = 4 biological replicates). (**C**) Representative images of sagittal OB sections from P0 mice. The cells undergoing apoptosis are immunostained with antibody against Cleaved-Caspase 3 (Activated-Caspase 3). (**D**) The density of Cleaved-Caspase 3^+^ cells in OB (unpaired Student’s *t* test, *n* = 3 biological replicates). Data information: Data are represented as mean ± SEM. n.s. not significant, *P* > 0.05. Scale bars: (**A**), 30 μm; (**C**) 300 μm and 30 μm in full-size images and zoomed areas.

**Figure EV4:**
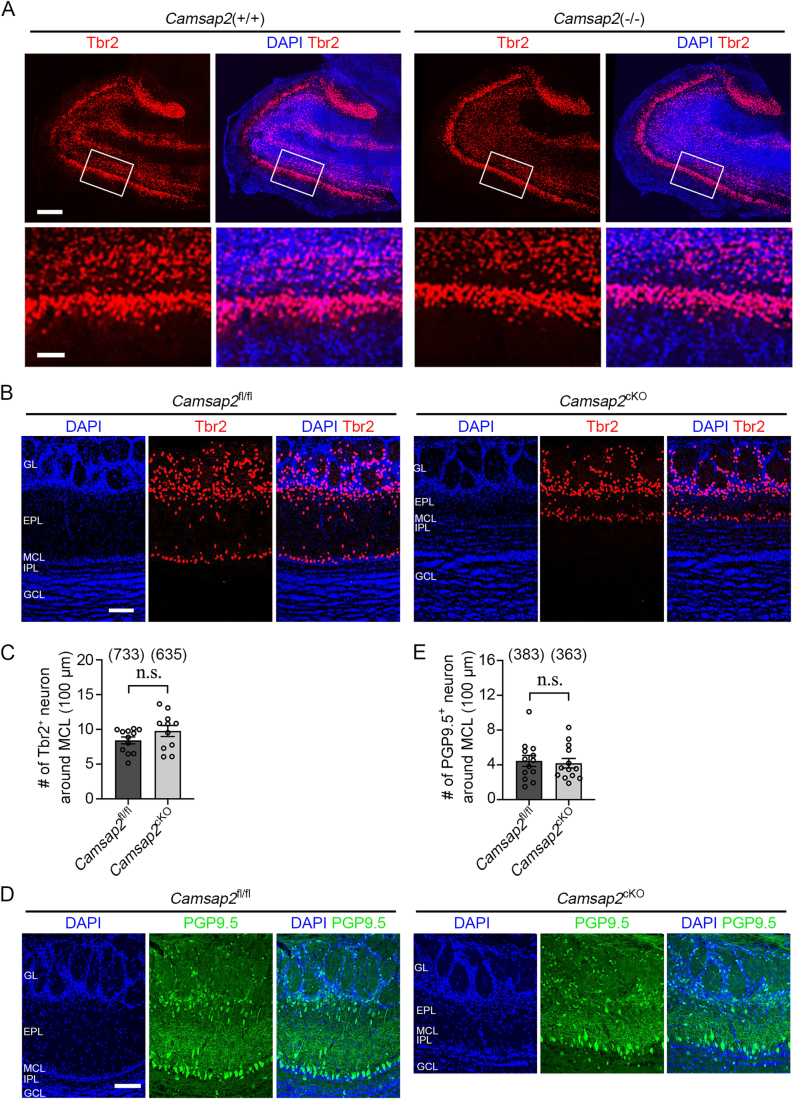
CAMSAP2 is dispensable for the migration of MCs. (**A**) Representative images of the sagittal sections of the OB from P0 mice. The MCs are immunostained with antibody against Tbr2. (**B**) Representative images of coronal sections of the OB of adult mice. The MCs are immunostained with. antibody against Tbr2. (**C**) Quantification of the number of Tbr2^+^ neurons around the MCL (unpaired Student’s *t* test, *n* = 3 biological replicates). (**D**) Representative images of coronal sections of the OB of adult mice. The MCs are immunostained with. antibody against PGP9.5. (**E**) Quantification of the number of PGP9.5 positive neurons around the MCL (unpaired Student’s *t* test, *n* = 3 biological replicates). Data information: Data are represented as mean ± SEM. n.s. not significant, *P* > 0.05. Scale bars: (**A**) 200 μm and 50 μm in full-size images and zoomed areas; (**B**, **D**) 400 μm.

The development of MCs is composed of two main stages: neurogenesis, migration and neurite extension in the embryonic stage, and dendrite remodeling (pruning and stabilization) in the first postnatal week (Fig. 4A) (Blanchart et al, 2006; Hinds, 1968; Imai, 2014; Imamura et al, 2011; Ravi et al, 2017). To determine which developmental stage CAMSAP2 participates in, we first analyzed the proliferation and migration of MCs. The fraction of Pax6^+^BrdU^+^/Pax6^+^ neuron was equivalent (Fig. EV3A,B), moreover, the density of MCs in the MCL was also unchanged (Fig. EV4B–E), suggesting that the neurogenesis of MCs is normal in CAMSAP2 deficient mice. We also excluded the possibility that deficiency of CAMSAP2 leads to the defects of apoptosis or migration in *Camsap2* KO or cKO mice (Figs. EV3C,D and EV4). However, the boundary of the MCL became obscure (Fig. EV4B,D). This result hinted that CAMSAP2 may participate in the process of dendrite remodeling, as the soma of MCs is aligned to form the MCL during this process (Ravi et al, 2017).

To verify our hypothesis, we first analyzed the distribution pattern of CAMSAP2 in the OB at the postnatal stage. CAMSAP2 showed high intensity in the EPL during remodeling, but this regional preference disappeared as the remodeling process finished in P7 (Fig. 4B). To further confirm that CAMSAP2 functions in regulating the remodeling of apical dendrites, the AAV virus was injected at E14.5 as before (Fig. 3E), while the morphology of MCs was analyzed at P0 and P7, the start and end of the pruning process. Since MCs at P0 do not form the typical mature morphology, we cannot divide the MCs into categories as before (Fig. 3G). For this reason, we counted the number of apical dendrites crossing the GL/EPL boundary (Fig. 4C,E), as these dendrites participate in the subsequent remodeling procedure. We found that the intersection numbers between CAMSAP2 deficient MCs and WT MCs are comparable at P0, while CAMSAP2 deficient MCs have more intersections at P7 (Fig. 4C–F). Besides, *Camsap2* deletion also decreases the proportion of MCs with single crossings at P7 (Fig. 4G), and CAMSAP2 deficient MCs tend to project into glomeruli that are further away in distance (Figs. 3I and 4H). Even though MCs at P0 do not show any differences, we still cannot completely exclude the possibility that CAMSAP2 regulates the growth of dendrites before remodeling (Yau et al, 2014), as rAAV virus usually needs weeks to start its expression, which may miss the key stage of dendritic extension. However, *Camsap2*(−/−) mice show growth delay at postnatal day (Appendix Fig. S1E), suggesting that CAMSAP2 may have stronger function during the remodeling stage. Thus, our results indicate that CAMSAP2 is required for the remodeling process of MCs.

In this study, we reveal that CAMSAP2 is required for the male mice mating and olfaction. Furthermore, we found that CAMSAP2 participates in the morphogenesis of MC dendrites. Surprisingly, our group’s newly published work demonstrates that CAMSAP1 but not CAMSAP2 plays an essential role in male fertility via regulating the process of spermiogenesis (Hu et al, 2023). Thus, our studies in the nervous and reproductive systems suggest that mammalian development requires the sophisticated cooperation of CAMSAP family members. Thus, finely tuned MT networks regulated by CAMSAPs are critical for the survival of mammals.

Each type of OSN typically projects its axons to two glomeruli in the OB, and MCs in the OB extend a single apical dendrite to a glomerulus to establish the connection between OR and olfactory cortex in a spatiotemporal manner (Imamura et al, 2011; Mombaerts et al, 1996). In this study, we observed that knocking out *Camsap2* resulted in abnormal morphology of MC dendrites, including multiple apical dendrites or no apical dendrites extended into EPL. All of these defects would be predicted to disrupt the wiring of the olfactory circuit and impair the detection or discrimination of chemical cues. In the OB, each glomerulus is innervated by apical dendrites from ~20 MCs (Imai, 2014; Ravi et al, 2017). These MCs constitute a group of sister MCs and code information about odor in a non-redundant manner (Dhawale et al, 2010). Though half of MCs had single apical dendrites when *Camsap2* was knocked out in those neurons, it was apparent that the projections of their apical dendrites were severely disrupted (Fig. 3F,I,J). This aberration leads to some adjacent MCs receiving different input while some non-adjacent MCs receive the same input, and these organizational mistakes may be harmful to odor information processing and eliciting behavior. Thus, the defects in the morphogenesis of MCs may contribute to the olfactory deficits observed in *Camsap2* KO mice, but that other (as yet unassessed) differences in the KO olfactory system may also play an important role. In our future work, we will try to design more experiments to explain it.

How do the MC dendrites find the right glomerulus to construct the precise circuit? Recent works show that Rac1 and RhoA regulate the MC dendrite remodeling in an activity-dependent manner (Aihara et al, 2021; Fujimoto et al, 2023). Interestingly, our previous study showed that CAMSAP3, the homolog of CAMSAP2, inhibits the activity of RhoA via GEF-H1 and this function is related to the detyrosination of MTs (Nagae et al, 2013). Since CAMSAP2 and CAMSAP3 are both responsible for the detyrosination of MTs (Tanaka et al, 2012), we propose that CAMSAP2 may responds to some signal to remodel dendrites through regulating the activity of RhoA. However, this hypothesis needs further investigation. *Camsap2* KO mice have thinner EPL and some of MCs lack connection with glomerulus, these hint that CAMSAP2 participates in multiple processes of dendrite remodeling, such as growth, pruning and stabilizing. Furthermore, our results suggest that *Camsap2* KO mice can be used as a model to study the molecular mechanism of dendrite remodeling. Although technical limitations constrained our exploration of the molecular mechanism, we are optimistic about uncovering the intricate role of CAMSAP2 as a mediator between remodeling signaling and the MT network. The potential breakthroughs awaiting us in future research hold the promise of shedding light on this captivating relationship.

For sexually reproducing species, male and female behavioral patterns are different in copulatory stages, with mounting, intromission and ejaculation performed by the male. For this reason, *Camsap2*(-/-) females could successfully birth pups in the mating assay. *Camsap2* KO mice did not display any normal innate responses to pheromonal and non-pheromonal odorants, suggesting that the function of the main olfactory bulb and the accessory olfactory bulb are both impaired. Furthermore, we found that deletion of *Camsap2* resulted in significantly thinner EPL in all six regions of the OB (Fig. EV2A–C). This suggests that CAMSAP2 may function in the remodeling of MCs in all areas other than some specific regions of OB. If this is the case, one would expect that the deletion of *Camsap2* would cause total loss of general olfaction ability. Indeed, our observations support this conclusion (Fig. 2). Thus, our results demonstrate a vital role of CAMSAP2 in the development of the olfactory system.

## Methods

### Animals

All animal experiments were approved by the Animal Center of the Institute of Genetics and Developmental Biology (IGDB), Chinese Academy of Sciences, and were conducted in accordance with the IACUC guidelines at IGDB. Mice were maintained in specific pathogen-free conditions in the animal facility and housed on a 12-h light/12-h dark cycle with ad libitum access to food and water. The *Camsap2*(+/−) and *Camsap2*^fl/+^ mice models were created by Beijing Biocytogen. Nestin-Cre mouse strain was gifted by Dr. Shilai Bao (IGDB). Unless stated otherwise, mice of both genders were used and their ages were indicated in the Figures or the legends. For timed pregnancy, mid-day when the vaginal plug was identified was calculated as embryonic day 0.5 (E0.5).

### Immunofluorescence

Immunostaining tissue was performed as described before(Zhou et al, 2020), with following antibodies: anti-CAMSAP2 (1:500, Proteintech, 17880-1-AP), anti-Tbr2 (1:1000, Abcam, ab183991), anti-NeuN (1:1000, Abcam, ab104224), anti-NF165 (1:300, DSHB, 2H3), anti-GFP (1:500, Invitrogen, A10262; 1:500, MBL, 598), anti-RFP (1:500, Chromotek, 5F8), anti-Pax6 (1:1000, BioLegend, 901301), anti-BrdU (1:1000, Abcam, ab6326), anti-Cleaved-Caspase 3 (1:800, CST, 9661), anti-PGP9.5 (UCH-L1, 1:200, Santa Cruz, sc-271639). Alexa fluor-conjugated secondary antibodies (Invitrogen) were used at 1:1000 dilution. For some antibodies, eg. Tbr2, the antigen retrieval was performed by steaming samples in a 98 °C antigen retrieval solution for 5 min and leaving samples in antigen retrieval solution to cool down to room temperature, the subsequent procedure was performed as described before (Zhou et al, 2020).

For BrdU labeling, pregnant females received a single intraperitoneal injection of BrdU (Sigma-Aldrich, 50 μg/g) at E11.5. Mice were sacrificed after injection for 2 h and fixed with PFA. The cryosections of the brain were incubated in HCl for appropriate times to denature DNA and then neutralized in 0.1 M borate buffer (pH 8.5). Subsequently, the section was immunostained with anti-Pax6 and BrdU antibodies following the standard protocol.

### Clearing with BABB

PFA-fixed brains were rinsed in PBS and embedded in 3% agarose gel. Samples were then sliced by a microslicer (Compresstome VF-300-0Z, Precisionary) at 300–400 μm thickness for P7 (Fig. 4D) and P21-P23 (Fig. 3F) mice. The clearing procedure was described previously (Erturk et al, 2011). Briefly, tissues were successively incubated in 50% and 80% tetrahydrofuran (THF) for 30 min. After three times in 100% THF for 30 min each, tissues were incubated in 100% dichloromethane for 20 min. Finally, the tissues were submerged in a mixture of BABB (benzyl alcohol and benzyl benzoate at a ratio of 1:2), as the tissues are thin, they were transparent in minutes and the tissue could be mounted on slides with BABB for confocal imaging.

To get higher signal of mScarlet positive (mScarlet^+^) neurons, we used the following protocol to stain the tissue with an anti-RFP antibody. The OB sections were permeabilized with wash buffer (1% Triton X-100 in PBS) three times for 30 min each, and after blocking with blocking solution (10% FBS and 5% BSA in wash buffer) O/N at 4 °C, the sections were incubated with primary antibodies at 4 °C for 2–4 days on a shaker. Then, the sections were washed with wash buffer and incubated with secondary antibody for 1–2 days on a shaker at 4 °C. Finally, the sections were washed with wash buffer, and washed with PBS three times after fixing with 4% PFA at RT for 20 min.

### H&E and Golgi-cox staining analysis

Testes were dissected from adult mice and fixed in 4% PFA O/N at 4 °C with rocking. After washing three times with PBS, the testes were stored in 70% ethanol until the process for paraffin, and the paraffin sections were placed on slides for H&E staining analysis.

For Golgi-cox staining, 2 months brains were stained using FD Rapid Golgi Stain Kit (FD Neurotechnologies PK401A) according to the manufacturer’s suggestions. OBs were sectioned at 100 μm and placed onto gelatin-coated microscope slides.

### Immunoblotting

Immunoblotting was performed as described before (Zhou et al, 2020), with the following antibodies: anti-CAMSAP1 (1:1000, Sigma, HPA024161), anti-CAMSAP2 (1:1000, Sigma, HPA027302), anti-CAMSAP3 (1:1000, homemade), anti-tubulin (1: 20,000, Sigma, T6074).

### Hormones

To generate estrus females, bilateral ovariectomy was carried out in 6–8 weeks-old female C57BL/6N mice. Ovariectomized females were allowed to recover for 3 weeks, the estradiol benzoate was subcutaneously injected at 48 h (10 μg in 100 μL sesame oil) and 24 h (5 μg in 50 μL sesame oil) before experiments, and progesterone (50 μg) was subcutaneously injected 4–6 h before experiments.

For analyzing the titers of serum sexual hormones, orbital blood was collected from adult males using a capillary tube, and the serum testosterone and estradiol levels were measured by Beijing North Institute of Biotechnology Co., Ltd.

### Infertility characterization

We first analyzed the male fertility by counting the pups produced by males. Briefly, sexually mature male mice were caged with 2-month-old WT C57BL/6N females for 4 months, the number of litters was counted. Plugging is also defined as a phenotype of male fertility, as it indicates mice mating behavior. WT males were paired with WT or *Camsap2* KO females, *Camsap2* KO males were paired with WT females, *Camsap2*^fl/fl^, and *Camsap2*^cKO^ males were paired with WT females for 5–6 days, and the days for appearance of the copulatory plug were counted. We caged the male with females at night and separated them in the morning.

Sperm were isolated from the epididymis and incubated in 37 °C pre-warmed PBS. Sperm motility was assessed using computer-assisted sperm analysis (Version.12 CEROS, Hamilton Thorne Research, Beverly, MA, USA). IVF was performed with 4-week-old C57BL/6N female, mice were superovulated by intraperitoneal (i.p.) injection with 10 IU pregnant mare serum gonadotropin (PMSG) followed 48 h later by 10 IU human chorionic gonadotropin (HCG). Cumulus-intact eggs were released from the oviducts 14–16 h after HCG injection and transferred to 200 μL drops of HTF medium. Sperm were obtained from the cauda epididymides of adult WT and *Camsap2* KO littermates and capacitated for 1 h in 200 μL drops of HTF medium. 5 μL sperm were added to the fertilization drop, and fertilization was allowed to proceed for 6 h. After washing out unbound sperm, the eggs were incubated O/N at 37 °C in a humidified atmosphere of 5% CO_2_. The inseminated eggs were examined for progression to the 2-cell stage, and the 2-cell embryos were transferred into the oviducts of pseudopregnant recipients.

### Behavioral assays

In all, 2–4-month-old male mice were used in behavioral assays.

#### Mating behavior

A mating behavior assay was initiated at least 1 h after the onset of the dark cycle, and the behaviors were recorded using an infrared-sensitive video recorder. The estrus female was introduced to the home cage of WT or *Camsap2* KO male, and videotaping for 30 min. The mating behavioral assay was repeated three times with different females at least three days apart. Mating behavior was scored according to the following criteria: nose-to-face, and nose-to-body are collectively as “non-anogenital”. Nose-to-anogenital contact is specifically defined as “anogenital”. “Mount” is scored when male places its forelimbs on the back of the estrus female.

#### Rotarod test

Mice were first acclimated to the rotarod for 2 min, followed by accelerating from 4 to 40 rpm, with the maximal test duration set at 5 min. The test was performed for consecutive 2 days and four trials per day with at least 60 min of rest between trials. The latency to fall off the rod was recorded.

#### Recording of USV

USV was detected using a condenser microphone (UltraSoundGate CM16, Avisoft Bioacoustics) that was connected to a converter (Avisoft Bioacoustics). The acoustic signals were recorded and analyzed by the software (Avisoft SASLab Pro). The vocalization of the single male was recorded for 2 min as background, and the vocalization was recorded for another 5 min after introducing the female. For young pups, they were separated from their parents and recorded for 5 min.

#### Urine preference test

Urine was collected from group-housed C57BL/6 N males or females. Urine samples from ten animals were pooled and stored at −80 °C. Before testing each day, aliquots of urine were thawed and diluted threefold for use. The subject mice were habituated to the clean cage for 30 min with two empty boxes on both sides, then 60 μL urine was dropped into the boxes and recorded for 5 min for analysis.

#### Territorial marking assay

Male mice were placed individually in cages for 3 days to habituate. After this habituation period, a piece of filter paper was placed on the bottom of the cage for 10 min to establish baseline urine marking. Removing the filter paper and placing a new paper under the cage. 100 μL of female urine was spotted into the center of the paper and left for 10 min. The paper was treated with ninhydrin spray and air dried until urine spots developed a purple color. Papers were scanned and the percentage of the paper marked by urine was determined using Image J software.

#### Buried food test

Male mice were deprived of food for 24 h and then placed in a new cage with sterile bedding. In total, 2 g of standard mouse chow was hidden from view under ~5 cm of bedding. We measured the time required for mice to find and begin to eat the food pellet. This experiment was performed in the daytime (7:00–19:00).

#### Defensive behavior

Defensive behavior was performed in a rectangle 60 × 40 cm open-field box. Two baffles with a 10 × 6 cm arch in the bottom to allow the mice entrance were inserted and divided the box into three chambers. Three sides of the hiding chamber were covered by black film and the remaining side was transparent to enable filming of mouse activity. Mice were habituated to the arena for 2 days. On day 3, a rat was deeply anesthetized by i.p. injection of sodium pentobarbital and placed in the rat chamber with its paws and snout facing the wall (see in Fig. 2D). Mice spent in each chamber were counted, and the total time of this experiment is 30 min. Avoidance behavior was defined as the amount of time mice spent inside the hiding chamber. Risk assessment used in this study is the flat-back/stretch-attend response. Initially, the mouse slowly approaches the rat with a lower head, then flattens its back while nearing the rat, resulting in an extended posture with a thrusted head; mostly, this behavior accompanies retreating/fleeing behavior.

### AAV injection

AAV injection was performed essentially as in utero electroporation described previously (Zhou et al, 2020). Briefly, mice were deeply anesthetized with sodium pentobarbital, and virus mixture (AAV2/9-TRE-Cre-WPRE, 2 × 10^9^ v.g./mL; AAV2/9-hSyn-DIO (tTA-P2A-mScarlet)-WPRE, 2 × 10^13^ v.g./mL) was injected into the embryonic OB at E14.5 with a glass microcapillary. Capillary was empirically inserted into the center of the OB, and each time, we injected 0.2 μL into a single OB base on the minimum scale of the syringe.

### Image processing

Images of sectioned brain slices and neurons were obtained through Zeiss confocal microscope Observer Z1 equipped with a spinning disk (Yokogawa CSU-1), confocal microscope LSM800, Olympus FV3000 confocal microscope, and Nikon confocal microscope (Eclipse Ti-C2). For H&E staining, images were obtained through Nikon Eclipse 80i equipped with DS-Ri1 microscope camera. For Golgi-cox staining, images were obtained via Olympus BX51 microscope.

### Statistical analyses

All the statistical details of experiments can be found in the figure legends. Graphpad Prism and Microsoft Excel were used for statistical analysis. All data collection and analysis for behavioral assays were blinded. The analysis for tissue sections was not blinded as the structural differences between WT and KO or cKO mice OB is so significant and it is easy to distinguish (narrowed EPL and blurred boundary of MCL). All the data are represented as mean ± SEM, and all conditions statistically different from the control are indicated (*P < 0.05, **P < 0.01, ***P < 0.001; n.s. not significant, *P* > 0.05). All experiments were performed with at least three biological replicates.

For normal immunostaining, at least two serial sections in the medial of OB were collected. For Golgi-cox staining (Fig. 3C) and BABB clearing tissue (Figs. 3F and 4D), all sections from the OB were collected. All images of immunostained tissue were analyzed using image J to quantify the cell number, morphology, and length of EPL manually.

## Supplementary information

Appendix

Peer Review File

Source data Fig. 1

Source data Fig. 2

Source data Fig. 3

Source data Fig. 4

Expanded View Figures

## Data Availability

This study includes no data deposited in external repositories. All data generated during this study are included in the main figures, expanded view, and appendix. The source data of this paper are collected in the following database record: biostudies:S-SCDT-10_1038-S44319-024-00166-x.

## References

[CR1] Aihara S, Fujimoto S, Sakaguchi R, Imai T (2021) BMPR-2 gates activity-dependent stabilization of primary dendrites during mitral cell remodeling. Cell Rep 35:109276 34161760 10.1016/j.celrep.2021.109276

[CR2] Akhmanova A, Hoogenraad CC (2015) Microtubule minus-end-targeting proteins. Curr Biol 25:R162–171 25689915 10.1016/j.cub.2014.12.027

[CR3] Asaba A, Hattori T, Mogi K, Kikusui T (2014) Sexual attractiveness of male chemicals and vocalizations in mice. Front Neurosci 8:231 25140125 10.3389/fnins.2014.00231 PMC4122165

[CR4] Baum MJ, Cherry JA (2015) Processing by the main olfactory system of chemosignals that facilitate mammalian reproduction. Horm Behav 68:53–64 24929017 10.1016/j.yhbeh.2014.06.003

[CR5] Blanchart A, De Carlos JA, Lopez-Mascaraque L (2006) Time frame of mitral cell development in the mice olfactory bulb. J Comp Neurol 496:529–543 16572431 10.1002/cne.20941

[CR6] Chen P, Hong W (2018) Neural circuit mechanisms of social behavior. Neuron 98:16–30 29621486 10.1016/j.neuron.2018.02.026 PMC6028944

[CR7] Dhawale AK, Hagiwara A, Bhalla US, Murthy VN, Albeanu DF (2010) Non-redundant odor coding by sister mitral cells revealed by light addressable glomeruli in the mouse. Nat Neurosci 13:1404–1412 20953197 10.1038/nn.2673 PMC3208311

[CR8] Dulac C, Wagner S (2006) Genetic analysis of brain circuits underlying pheromone signaling. Annu Rev Genet 40:449–467 16953793 10.1146/annurev.genet.39.073003.093937

[CR9] Erturk A, Mauch CP, Hellal F, Forstner F, Keck T, Becker K, Jahrling N, Steffens H, Richter M, Hubener M et al (2011) Three-dimensional imaging of the unsectioned adult spinal cord to assess axon regeneration and glial responses after injury. Nat Med 18:166–171 22198277 10.1038/nm.2600

[CR10] Fujimoto S, Leiwe MN, Aihara S, Sakaguchi R, Muroyama Y, Kobayakawa R, Kobayakawa K, Saito T, Imai T (2023) Activity-dependent local protection and lateral inhibition control synaptic competition in developing mitral cells in mice. Dev Cell 58:1221–1236.e1227 37290446 10.1016/j.devcel.2023.05.004

[CR11] Hendershott MC, Vale RD (2014) Regulation of microtubule minus-end dynamics by CAMSAPs and Patronin. Proc Natl Acad Sci USA 111:5860–5865 24706919 10.1073/pnas.1404133111 PMC4000804

[CR12] Hinds JW (1968) Autoradiographic study of histogenesis in the mouse olfactory bulb. II. Cell proliferation and migration. J Comp Neurol 134:305–322 5721257 10.1002/cne.901340305

[CR13] Ho KH, Jayathilake A, Mahircan Y, Nour A, Osipovich AB, Magnuson MA, Gu G, Kaverina I (2023) CAMSAP2 localizes to the Golgi in islet β-cells and facilitates Golgi-ER trafficking. iScience 26:105938 36718359 10.1016/j.isci.2023.105938 PMC9883185

[CR14] Hu W, Zhang R, Xu H, Li Y, Yang X, Zhou Z, Huang X, Wang Y, Ji W, Gao F et al (2023) CAMSAP1 role in orchestrating structure and dynamics of manchette microtubule minus-ends impacts male fertility during spermiogenesis. Proc Natl Acad Sci USA 120:e2313787120 37903275 10.1073/pnas.2313787120 PMC10636317

[CR15] Hull EM, Dominguez JM (2007) Sexual behavior in male rodents. Horm Behav 52:45–55 17499249 10.1016/j.yhbeh.2007.03.030 PMC1952538

[CR16] Imai T (2014) Construction of functional neuronal circuitry in the olfactory bulb. Semin Cell Dev Biol 35:180–188 25084319 10.1016/j.semcdb.2014.07.012

[CR17] Imamura F, Ayoub AE, Rakic P, Greer CA (2011) Timing of neurogenesis is a determinant of olfactory circuitry. Nat Neurosci 14:331–337 21297629 10.1038/nn.2754 PMC3046046

[CR18] Inokuchi K, Imamura F, Takeuchi H, Kim R, Okuno H, Nishizumi H, Bito H, Kikusui T, Sakano H (2017) Nrp2 is sufficient to instruct circuit formation of mitral-cells to mediate odour-induced attractive social responses. Nat Commun 8:15977 28731029 10.1038/ncomms15977 PMC5525001

[CR19] Jiang K, Hua S, Mohan R, Grigoriev I, Yau KW, Liu Q, Katrukha EA, Altelaar AF, Heck AJ, Hoogenraad CC et al (2014) Microtubule minus-end stabilization by polymerization-driven CAMSAP deposition. Dev Cell 28:295–309 24486153 10.1016/j.devcel.2014.01.001

[CR20] Khalaf-Nazzal R, Fasham J, Inskeep KA, Blizzard LE, Leslie JS, Wakeling MN, Ubeyratna N, Mitani T, Griffith JL, Baker W et al (2022) Bi-allelic CAMSAP1 variants cause a clinically recognizable neuronal migration disorder. Am J Hum Genet 109:2068–2079 36283405 10.1016/j.ajhg.2022.09.012 PMC9674946

[CR21] Kobayakawa K, Kobayakawa R, Matsumoto H, Oka Y, Imai T, Ikawa M, Okabe M, Ikeda T, Itohara S, Kikusui T et al (2007) Innate versus learned odour processing in the mouse olfactory bulb. Nature 450:503–508 17989651 10.1038/nature06281

[CR22] Lehti MS, Sironen A (2016) Formation and function of the manchette and flagellum during spermatogenesis. Reproduction 151:R43–54 26792866 10.1530/REP-15-0310

[CR23] Lin DM, Wang F, Lowe G, Gold GH, Axel R, Ngai J, Brunet L (2000) Formation of precise connections in the olfactory bulb occurs in the absence of odorant-evoked neuronal activity. Neuron 26:69–80 10798393 10.1016/s0896-6273(00)81139-3

[CR24] Lledo PM, Gheusi G, Vincent JD (2005) Information processing in the mammalian olfactory system. Physiol Rev 85:281–317 15618482 10.1152/physrev.00008.2004

[CR25] Luo W, Mizuno H, Iwata R, Nakazawa S, Yasuda K, Itohara S, Iwasato T (2016) Supernova: a versatile vector system for single-cell labeling and gene function studies in vivo. Sci Rep. 6:35747 27775045 10.1038/srep35747 PMC5075795

[CR26] Matsutani S, Yamamoto N (2000) Differentiation of mitral cell dendrites in the developing main olfactory bulbs of normal and naris-occluded rats. J Comp Neurol 418:402–410 10713569

[CR27] Meng W, Mushika Y, Ichii T, Takeichi M (2008) Anchorage of microtubule minus ends to adherens junctions regulates epithelial cell-cell contacts. Cell 135:948–959 19041755 10.1016/j.cell.2008.09.040

[CR28] Mombaerts P, Wang F, Dulac C, Chao SK, Nemes A, Mendelsohn M, Edmondson J, Axel R (1996) Visualizing an olfactory sensory map. Cell 87:675–686 8929536 10.1016/s0092-8674(00)81387-2

[CR29] Muroyama Y, Baba A, Kitagawa M, Saito T (2016) Olfactory sensory neurons control dendritic complexity of mitral cells via notch signaling. PLoS Genet 12:e1006514 28027303 10.1371/journal.pgen.1006514 PMC5189955

[CR30] Nagae S, Meng W, Takeichi M (2013) Non-centrosomal microtubules regulate F-actin organization through the suppression of GEF-H1 activity. Genes Cells 18:387–396 23432781 10.1111/gtc.12044

[CR31] Nyby J, Matochik JA, Barfield RJ (1992) Intracranial androgenic and estrogenic stimulation of male-typical behaviors in house mice (*Mus domesticus*). Horm Behav 26:24–45 1563726 10.1016/0018-506x(92)90029-u

[CR32] O’Donnell L (2014) Mechanisms of spermiogenesis and spermiation and how they are disturbed. Spermatogenesis 4:e979623 26413397 10.4161/21565562.2014.979623 PMC4581055

[CR33] Papes F, Logan DW, Stowers L (2010) The vomeronasal organ mediates interspecies defensive behaviors through detection of protein pheromone homologs. Cell 141:692–703 20478258 10.1016/j.cell.2010.03.037 PMC2873972

[CR34] Pomerantz SM, Nunez AA, Bean NJ (1983) Female behavior is affected by male ultrasonic vocalizations in house mice. Physiol Behav 31:91–96 6685321 10.1016/0031-9384(83)90101-4

[CR35] Pongrakhananon V, Saito H, Hiver S, Abe T, Shioi G, Meng WX, Takeichia M (2018) CAMSAP3 maintains neuronal polarity through regulation of microtubule stability. Proc Natl Acad Sci USA 115:9750–9755 30190432 10.1073/pnas.1803875115 PMC6166842

[CR36] Ravi N, Sanchez-Guardado L, Lois C, Kelsch W (2017) Determination of the connectivity of newborn neurons in mammalian olfactory circuits. Cell Mol Life Sci 74:849–867 27695873 10.1007/s00018-016-2367-y PMC11107630

[CR37] Robinson AM, Takahashi S, Brotslaw EJ, Ahmad A, Ferrer E, Procissi D, Richter CP, Cheatham MA, Mitchell BJ, Zheng J (2020) CAMSAP3 facilitates basal body polarity and the formation of the central pair of microtubules in motile cilia. Proc Natl Acad Sci USA 117:13571–13579 32482850 10.1073/pnas.1907335117 PMC7306751

[CR38] Rosenbaum JL, Witman GB (2002) Intraflagellar transport. Nat Rev Mol Cell Biol 3:813–825 12415299 10.1038/nrm952

[CR39] Schoeller EL, Clark DD, Dey S, Cao NV, Semaan SJ, Chao LW, Kauffman AS, Stowers L, Mellon PL (2016) Bmal1 is required for normal reproductive behaviors in male mice. Endocrinology 157:4914–4929 27704948 10.1210/en.2016-1620 PMC5133342

[CR40] Tanaka N, Meng W, Nagae S, Takeichi M (2012) Nezha/CAMSAP3 and CAMSAP2 cooperate in epithelial-specific organization of noncentrosomal microtubules. Proc Natl Acad Sci USA 109:20029–20034 23169647 10.1073/pnas.1218017109 PMC3523837

[CR41] Taniguchi K, Saito H, Okamura M, Ogawa K (1993) Immunohistochemical demonstration of protein gene product 9.5 (PGP 9.5) in the primary olfactory system of the rat. Neurosci Lett 156:24–26 8414184 10.1016/0304-3940(93)90430-s

[CR42] Togashi K, Tsuji M, Takeuchi S, Nakahama R, Koizumi H, Emoto K (2020) Adeno-associated virus-mediated single-cell labeling of mitral cells in the mouse olfactory bulb: insights into the developmental dynamics of dendrite remodeling. Front Cell Neurosci 14:572256 33362468 10.3389/fncel.2020.572256 PMC7756102

[CR43] Wu J, de Heus C, Liu Q, Bouchet BP, Noordstra I, Jiang K, Hua S, Martin M, Yang C, Grigoriev I et al (2016) Molecular pathway of microtubule organization at the Golgi apparatus. Dev Cell 39:44–60 27666745 10.1016/j.devcel.2016.08.009

[CR44] Wu MV, Manoli DS, Fraser EJ, Coats JK, Tollkuhn J, Honda S, Harada N, Shah NM (2009) Estrogen masculinizes neural pathways and sex-specific behaviors. Cell 139:61–72 19804754 10.1016/j.cell.2009.07.036 PMC2851224

[CR45] Yau KW, van Beuningen SF, Cunha-Ferreira I, Cloin BM, van Battum EY, Will L, Schatzle P, Tas RP, van Krugten J, Katrukha EA et al (2014) Microtubule minus-end binding protein CAMSAP2 controls axon specification and dendrite development. Neuron 82:1058–1073 24908486 10.1016/j.neuron.2014.04.019

[CR46] Zhou Z, Xu H, Li Y, Yang M, Zhang R, Shiraishi A, Kiyonari H, Liang X, Huang X, Wang Y et al (2020) CAMSAP1 breaks the homeostatic microtubule network to instruct neuronal polarity. Proc Natl Acad Sci USA 117:22193–22203 32839317 10.1073/pnas.1913177117 PMC7486724

