# Supplementary material for: Deficiency of CAMSAP2 impairs olfaction and the morphogenesis of mitral cells
Source: EMBO Rep. 2024 Jun 5;25(7):7. doi: 10.1038/s44319-024-00166-x (PMC11239855; doi:10.1038/s44319-024-00166-x)
Supplement: Supplementary file 1 — Appendix [file 44319_2024_166_MOESM1_ESM.pdf]

APPENDIX

Deficiency of CAMSAP2 impairs olfaction and the morphogenesis of mitral cells

Zhengrong Zhou, Xiaojuan Yang, Aihua Mao, Honglin Xu, Chunnuan Lin, Mengge Yang, Weichang Hu, Jinhui Shao, Peipei Xu, Yuejia Li, Wenguang Li, Ruifan Lin, Rui Zhang, Qi Xie, Zhiheng Xu, Wenxiang Meng

Table of Contents

Appendix Figure S1. Necessary information about *Camsap2*(-/-) mice. 2

Appendix Figure S2. Necessary information about *Camsap2*^{cKO} mice. 3

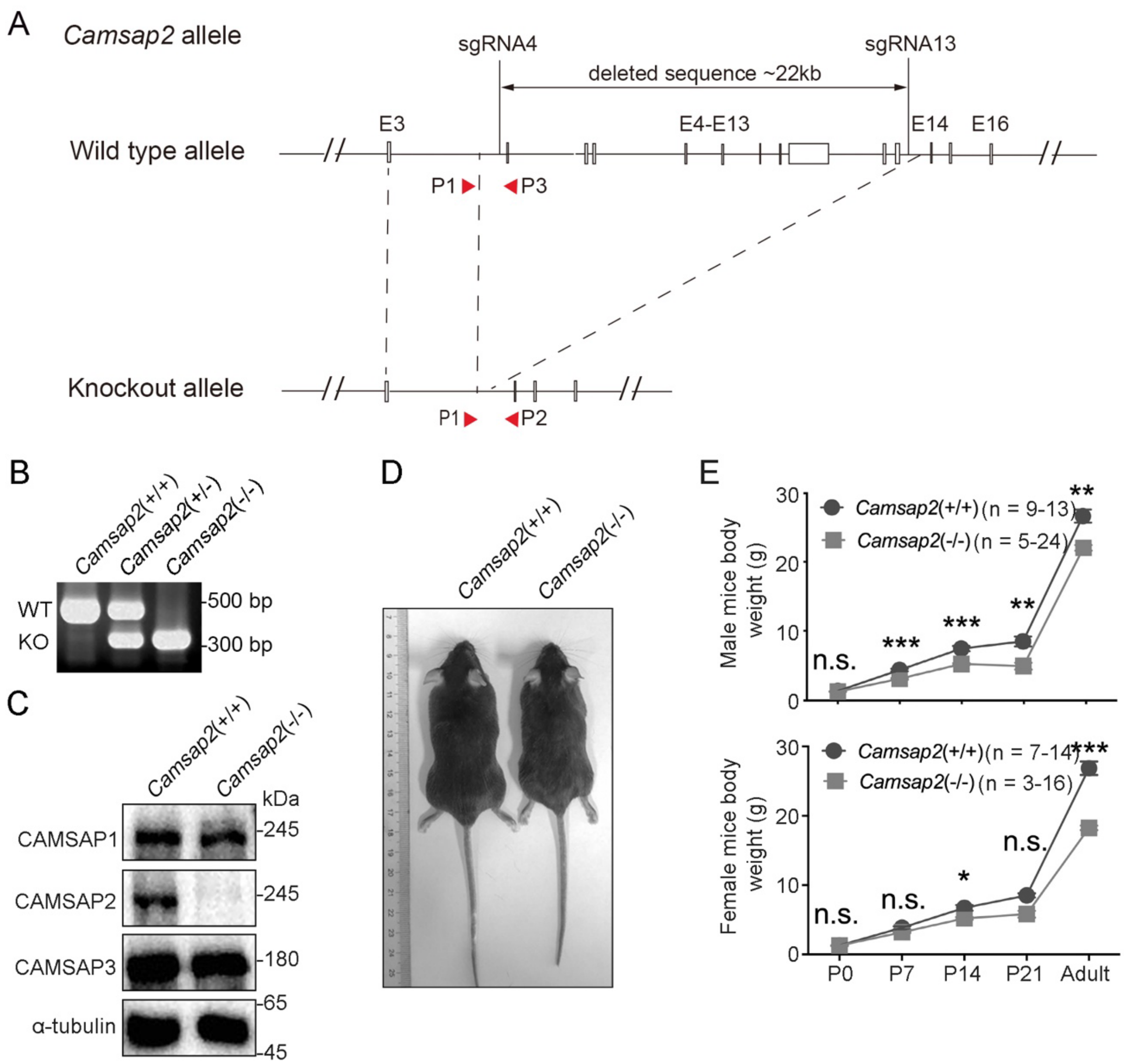

Appendix Figure S1. Necessary information about *Camsap2*(-/-) mice.

(**A**) The strategy of construction of *Camsap2*(-/-) mice. Exon 3 - exon 16 of *Camsap2* is shown.

(**B**) Genotypes of mice were verified by PCR.

(**C**) Western blot analysis of CAMSAPs in adult *Camsap2*(+/+) and *Camsap2*(-/-) mice brain.

(**D**) Image of *Camsap2*(+/+) and *Camsap2*(-/-) adult mice.

(**E**) The curves for the body weight of male mice (upper) and female mice (bottom) (two-way R-M ANOVA with Šídák's multiple comparisons test, male, n = 5 - 24 biological replicates, **p = 0.0043 or 0.0063, ***p = 0.0003 or < 0.0001; female, n = 3 - 16 biological replicates, *p = 0.029, ***p < 0.0001).

Data information: Data are represented as mean ± SEM.

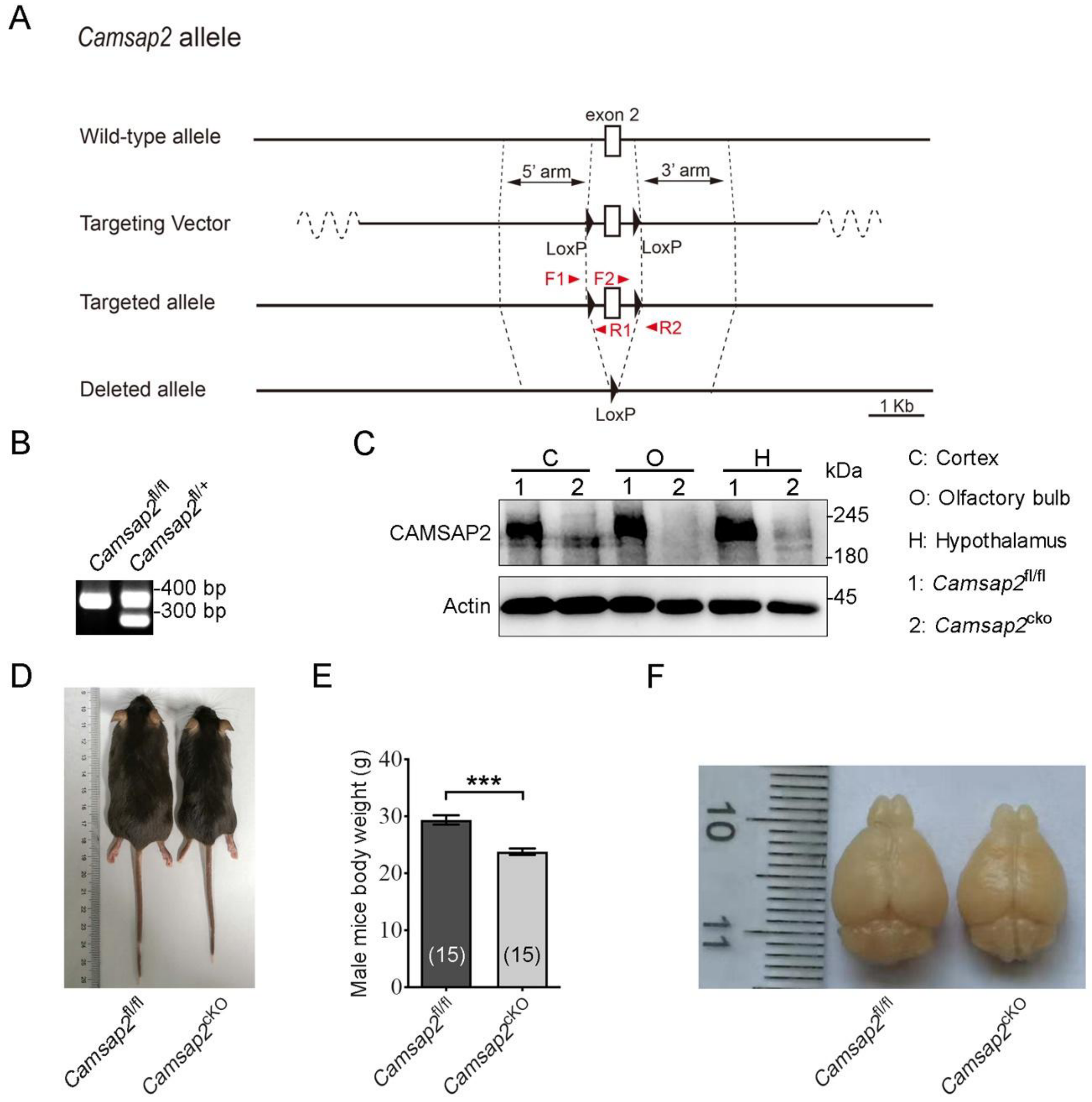

Appendix Figure S2. Necessary information about *Camsap2*^{cKO} mice.

(**A**) The strategy of construction of *Camsap2*^{cKO} mice.

(**B**) Genotypes of mice were verified by PCR.

(**C**) Western blot analysis of CAMSAP2 protein in *Camsap2*$^{\text{fl/fl}}$ and *Camsap2*^{cKO} adult mice brain.

(**D**) Image of *Camsap2*$^{\text{fl/fl}}$ and *Camsap2*^{cKO} adult mice.

(**E**) The body weight of adult male mice (unpaired student's t-test, n = 3 biological replicates, ***p = 7.65 $\times 10^{-6}$).

(**F**) Image of *Camsap2*$^{\text{fl/fl}}$ and *Camsap2*^{cKO} adult mice brain

Data information: Data are represented as mean ± SEM.
