# Supplementary figures and images for: Deficiency of CAMSAP2 impairs olfaction and the morphogenesis of mitral cells
Source: EMBO Rep. 2024 Jun 5;25(7):7. doi: 10.1038/s44319-024-00166-x (PMC11239855; doi:10.1038/s44319-024-00166-x)

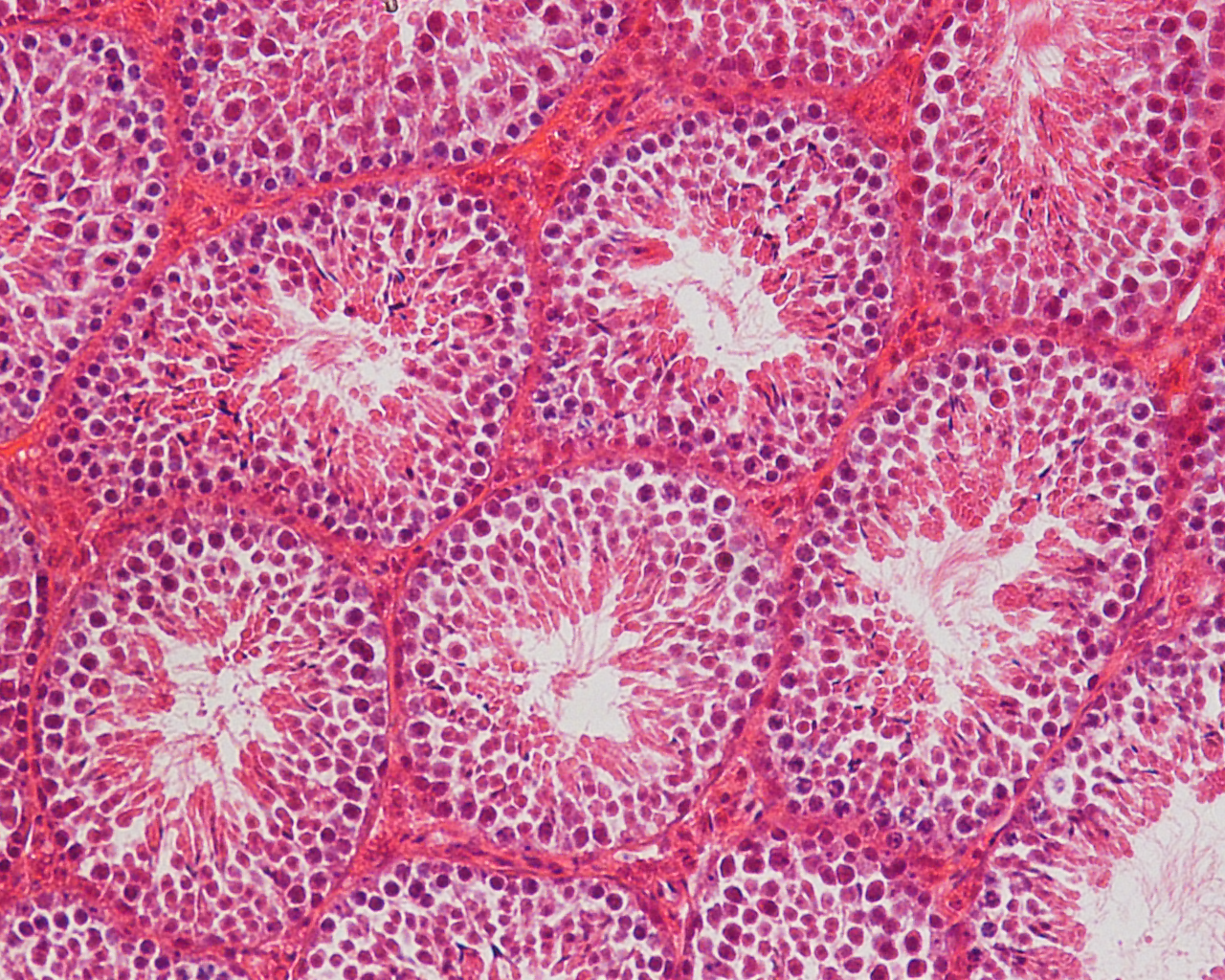

Supplement: Supplementary file 3 — Source data Fig. 1 [file 44319_2024_166_MOESM3_ESM.zip › Source Data for Figure 1/1B/ko-testis-20x-2.tif]

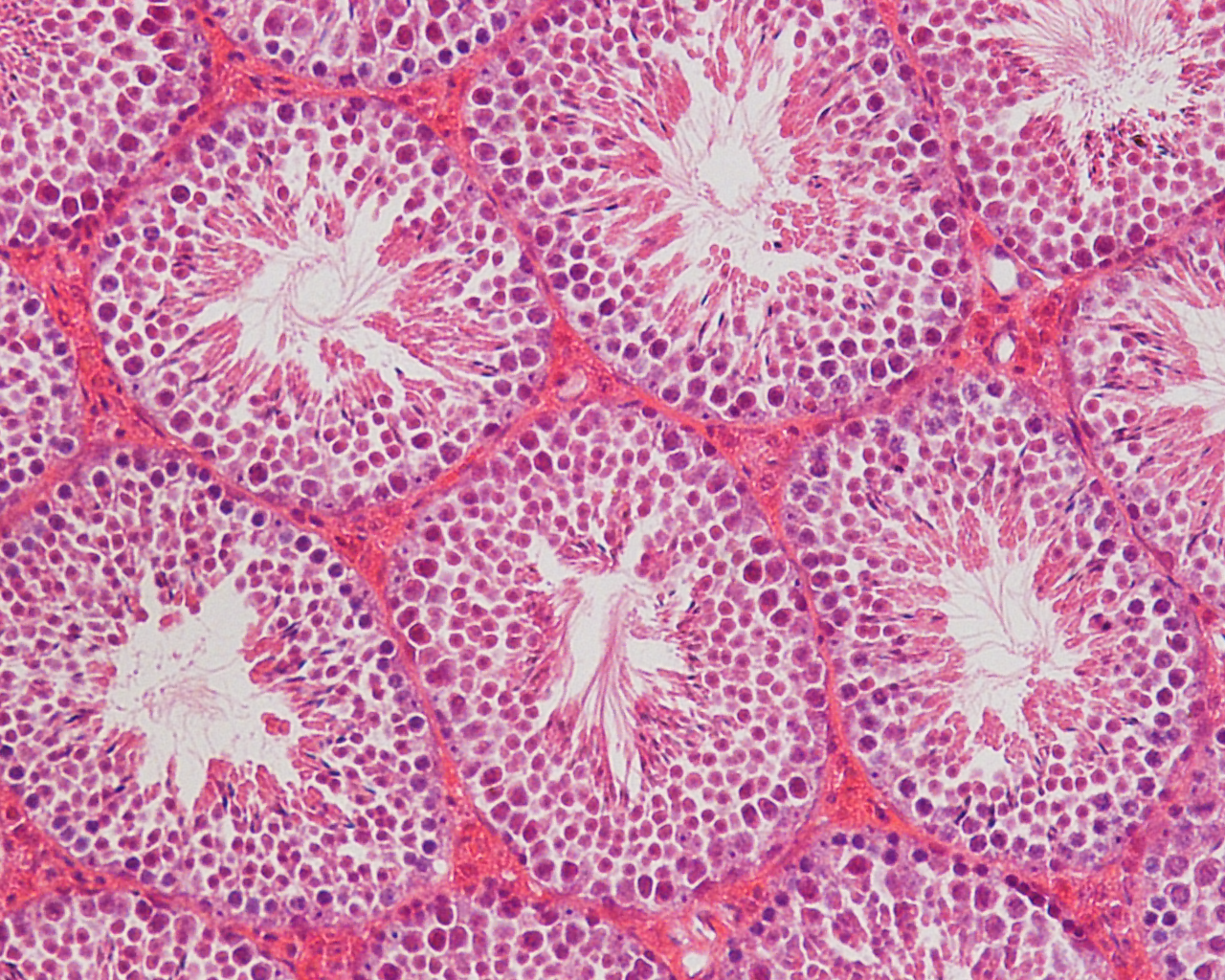

Supplement: Supplementary file 3 — Source data Fig. 1 [file 44319_2024_166_MOESM3_ESM.zip › Source Data for Figure 1/1B/wt-testis-20x-2-2.tif]

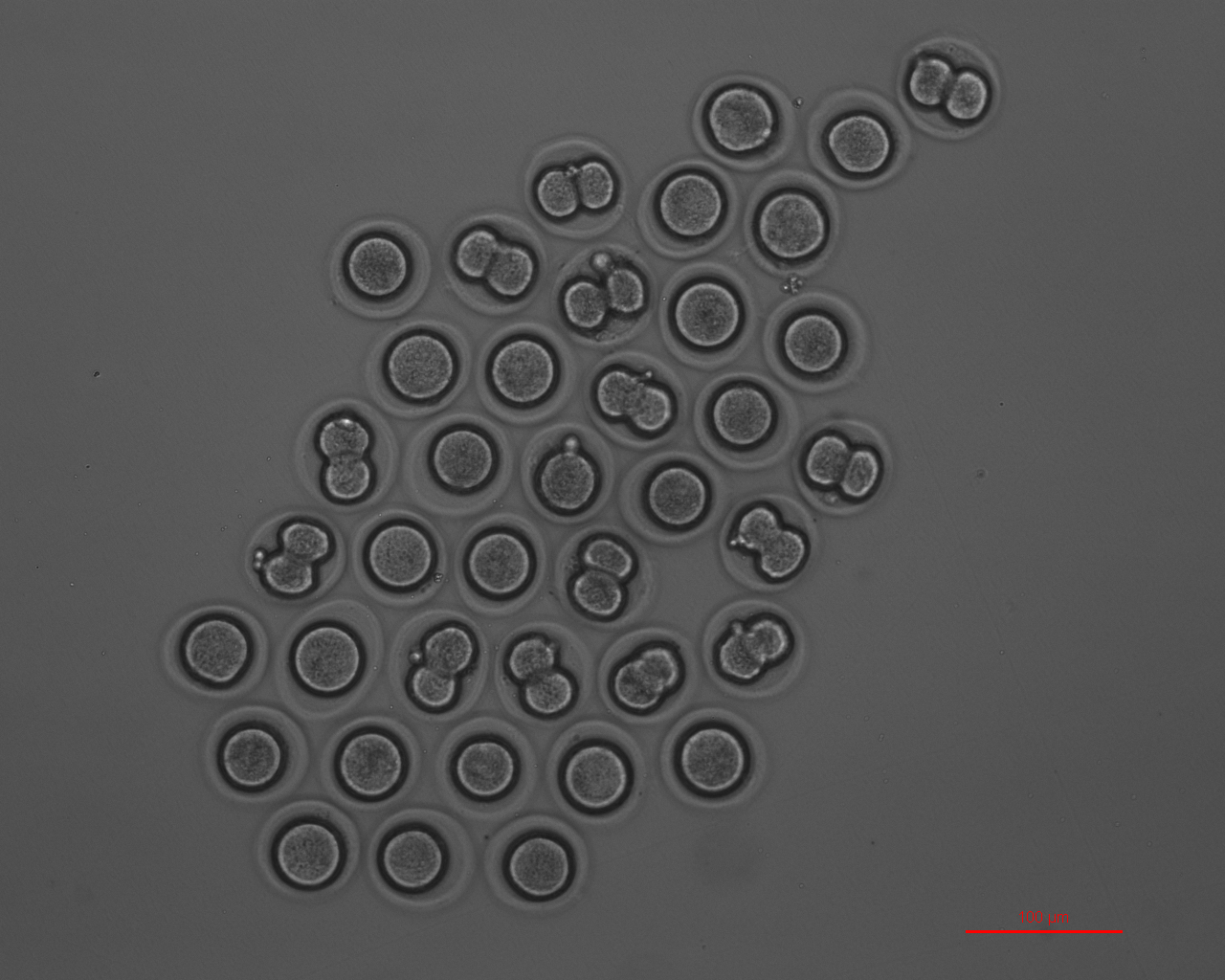

Supplement: Supplementary file 3 — Source data Fig. 1 [file 44319_2024_166_MOESM3_ESM.zip › Source Data for Figure 1/1D/WT_1-3(170922).tif]

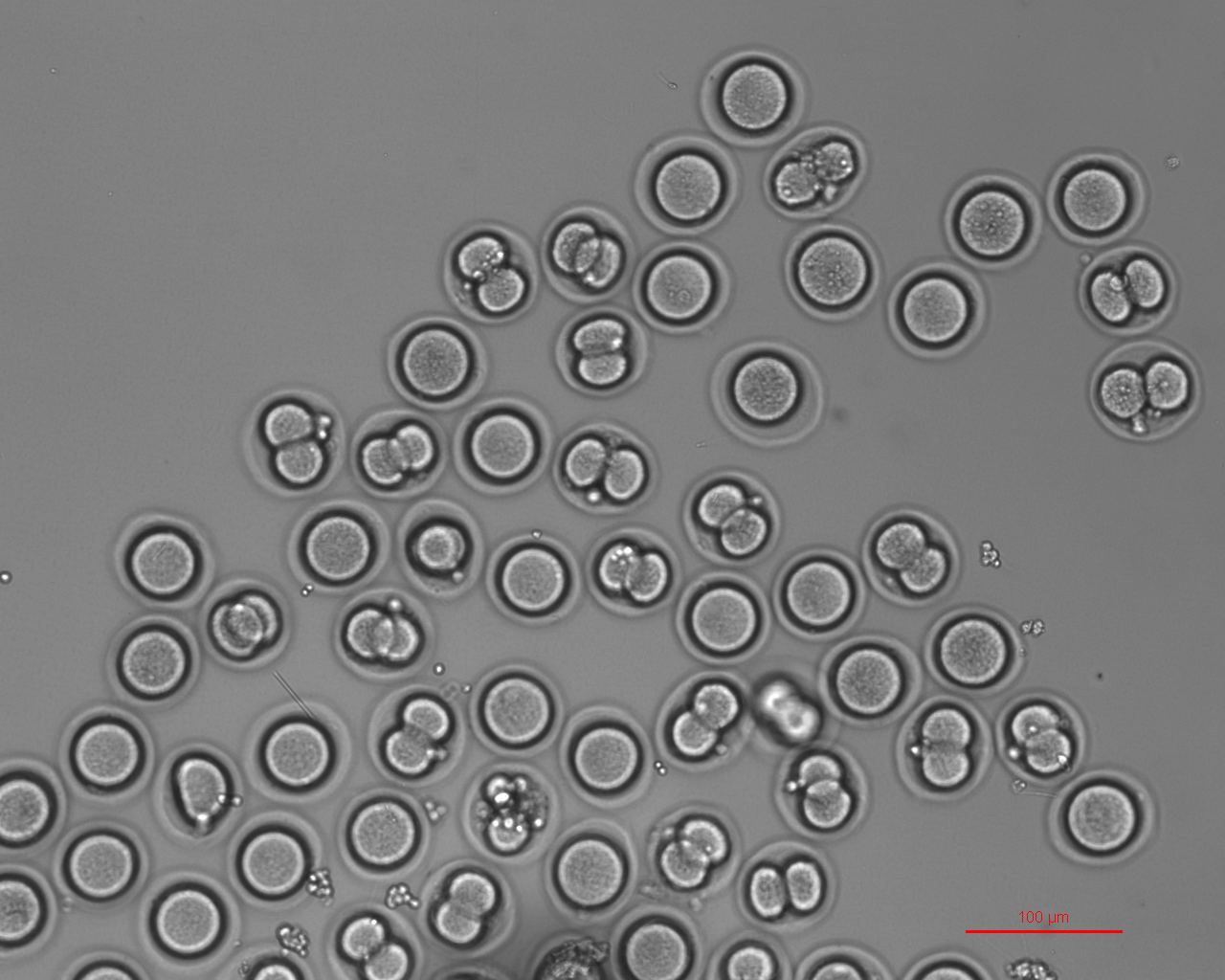

Supplement: Supplementary file 3 — Source data Fig. 1 [file 44319_2024_166_MOESM3_ESM.zip › Source Data for Figure 1/1D/KO_2#-10x-3(170503).tif]

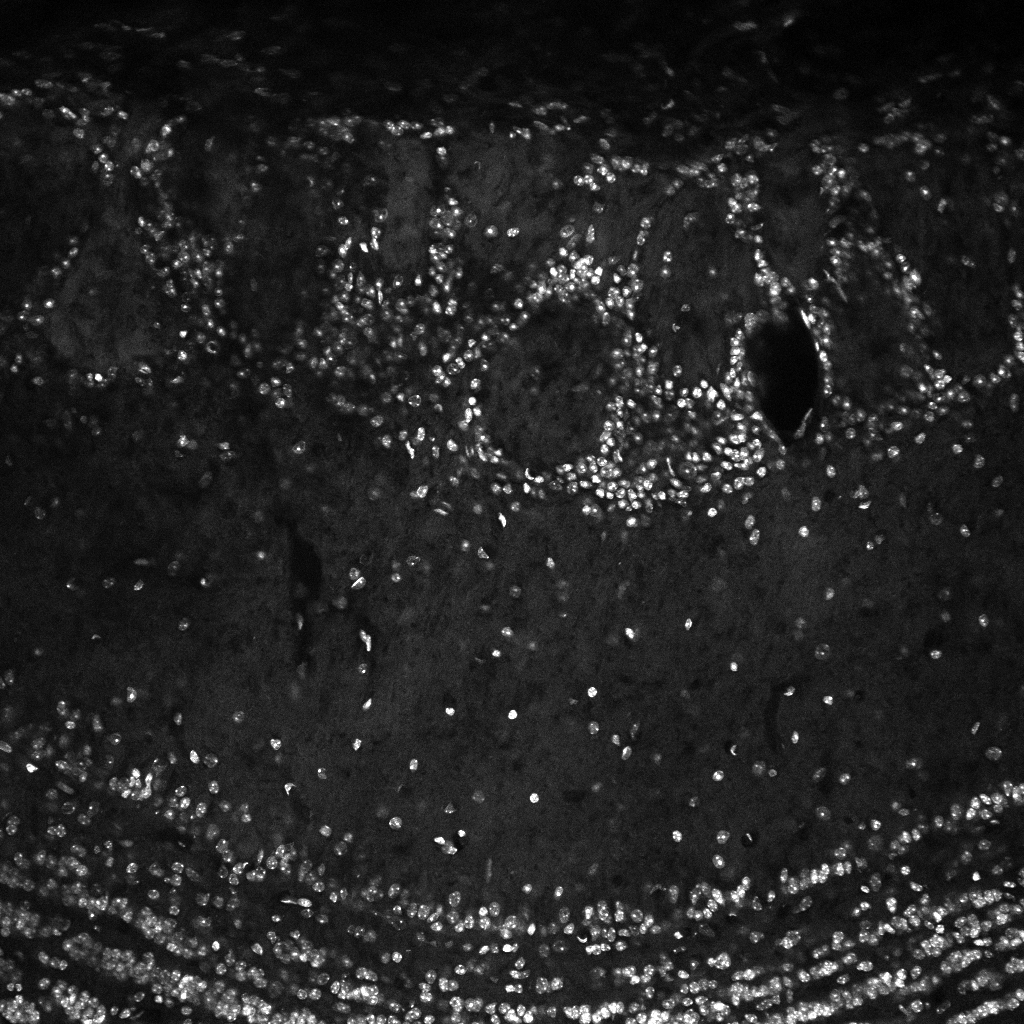

Supplement: Supplementary file 5 — Source data Fig. 3 [file 44319_2024_166_MOESM5_ESM.zip › Source Data for Figure 3/3B/20201001_4M_C2flox_PGP9.5DAPI_WT_02.tif]

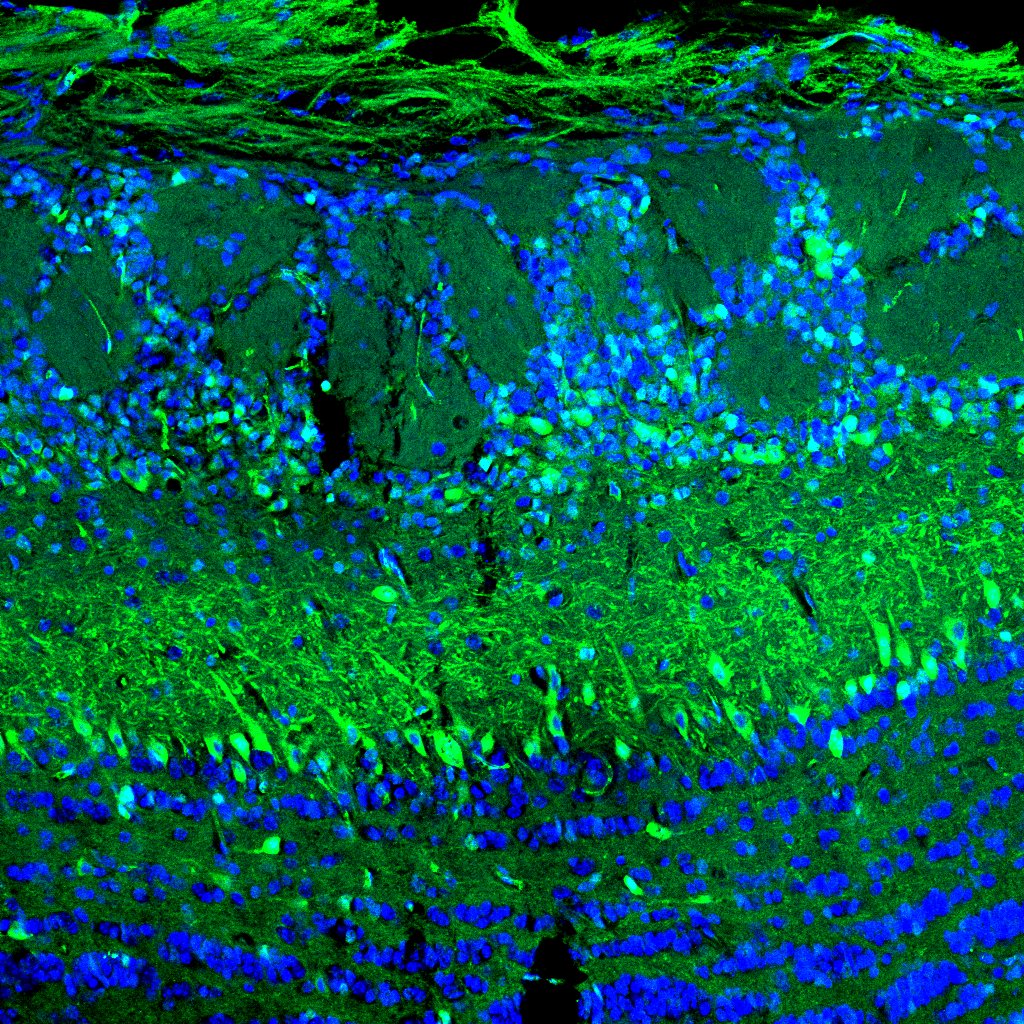

Supplement: Supplementary file 5 — Source data Fig. 3 [file 44319_2024_166_MOESM5_ESM.zip › Source Data for Figure 3/3B/20201001_4M_CreC2flox_PGP9.5DAPI_cKO_02.jpg]

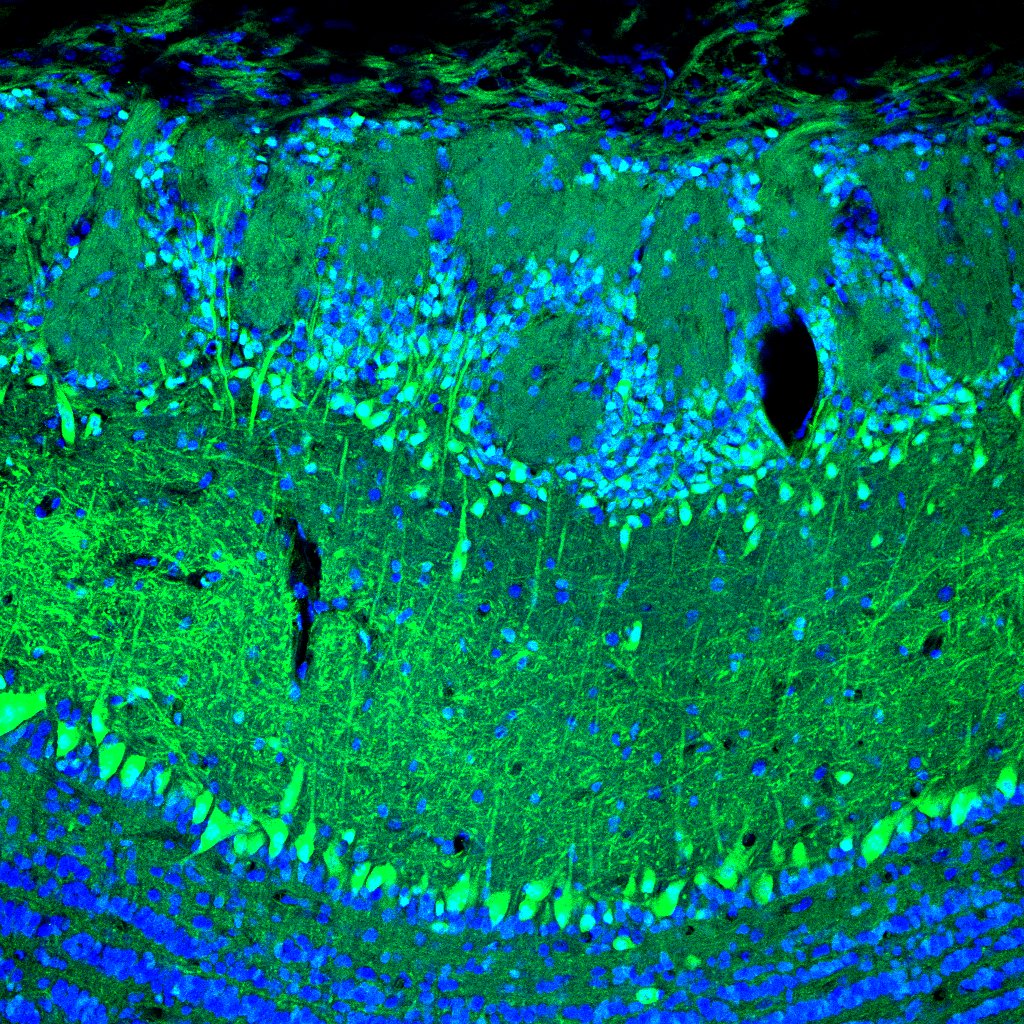

Supplement: Supplementary file 5 — Source data Fig. 3 [file 44319_2024_166_MOESM5_ESM.zip › Source Data for Figure 3/3B/20201001_4M_C2flox_PGP9.5DAPI_WT_02.jpg]

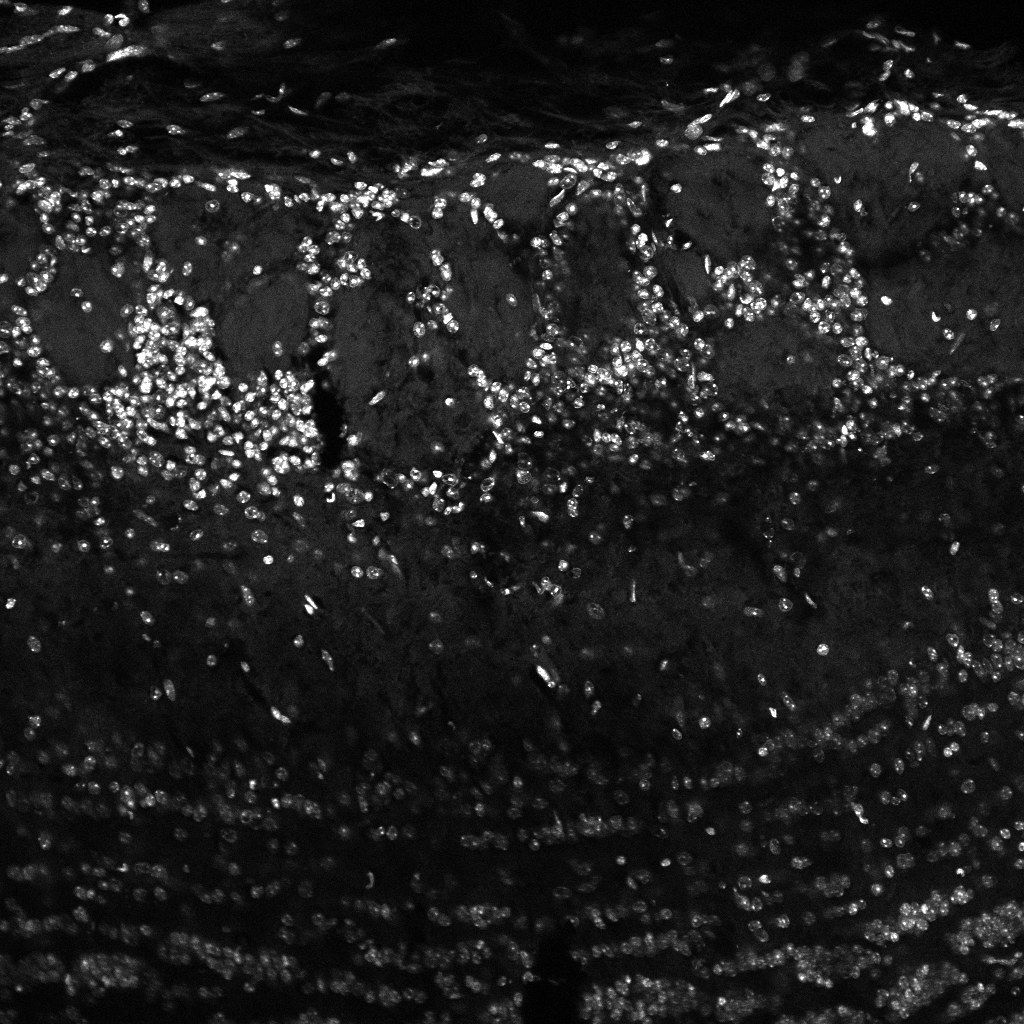

Supplement: Supplementary file 5 — Source data Fig. 3 [file 44319_2024_166_MOESM5_ESM.zip › Source Data for Figure 3/3B/20201001_4M_CreC2flox_PGP9.5DAPI_cKO_02.tif]

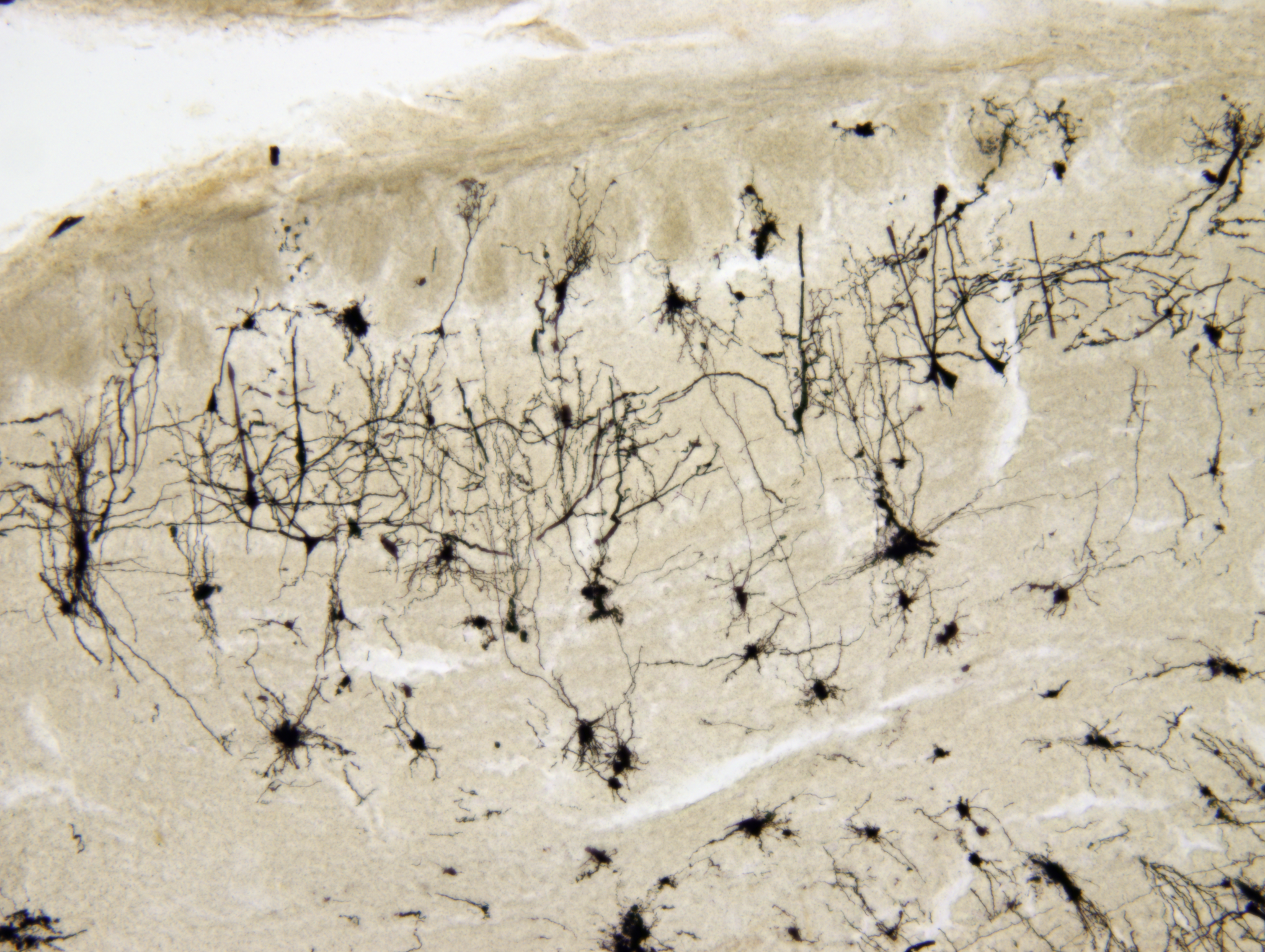

Supplement: Supplementary file 5 — Source data Fig. 3 [file 44319_2024_166_MOESM5_ESM.zip › Source Data for Figure 3/3C/SUM_flox-12d-adult-10x-1.tif]

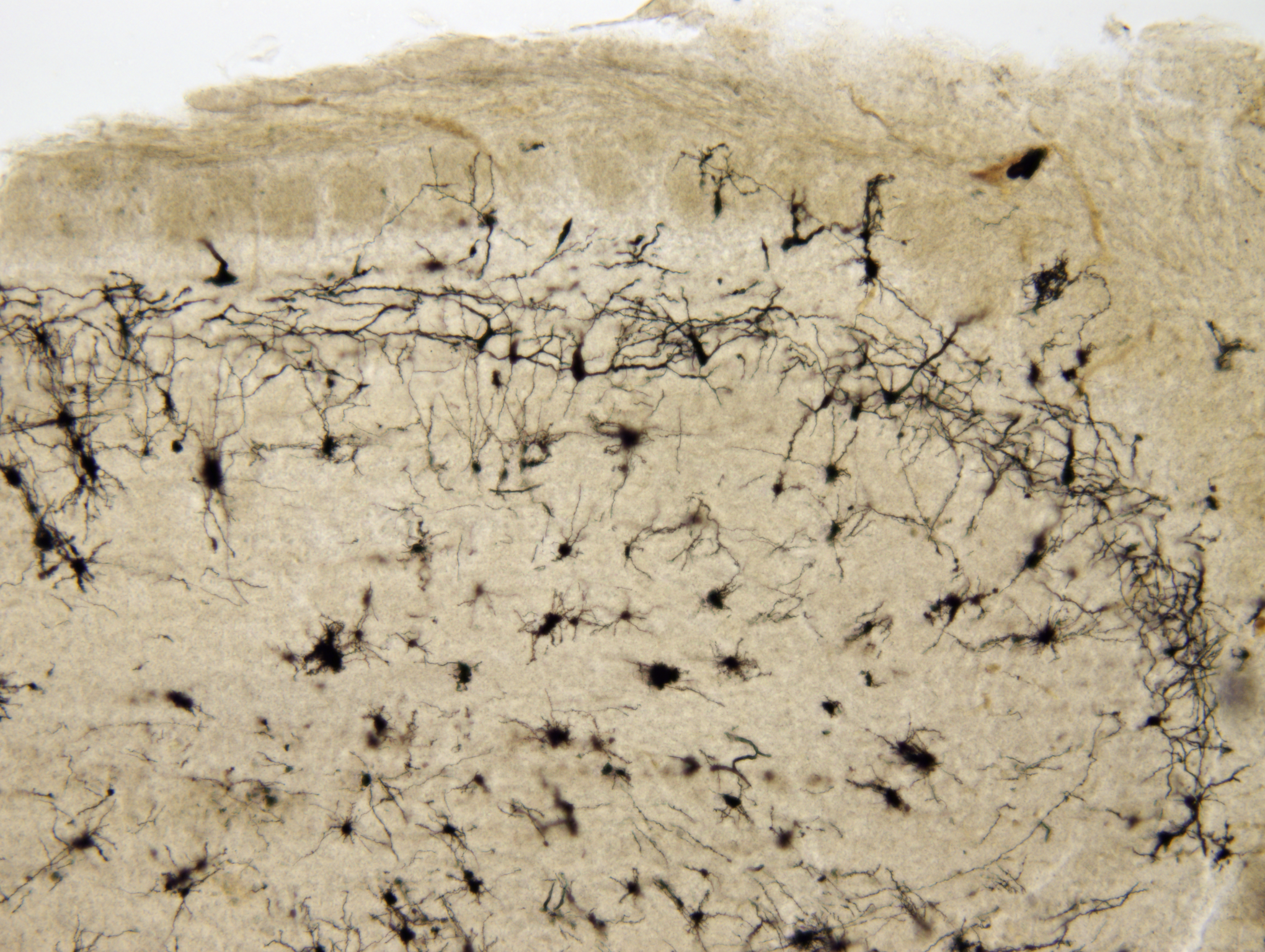

Supplement: Supplementary file 5 — Source data Fig. 3 [file 44319_2024_166_MOESM5_ESM.zip › Source Data for Figure 3/3C/SUM_cko-12d-adult-10x-1.tif]

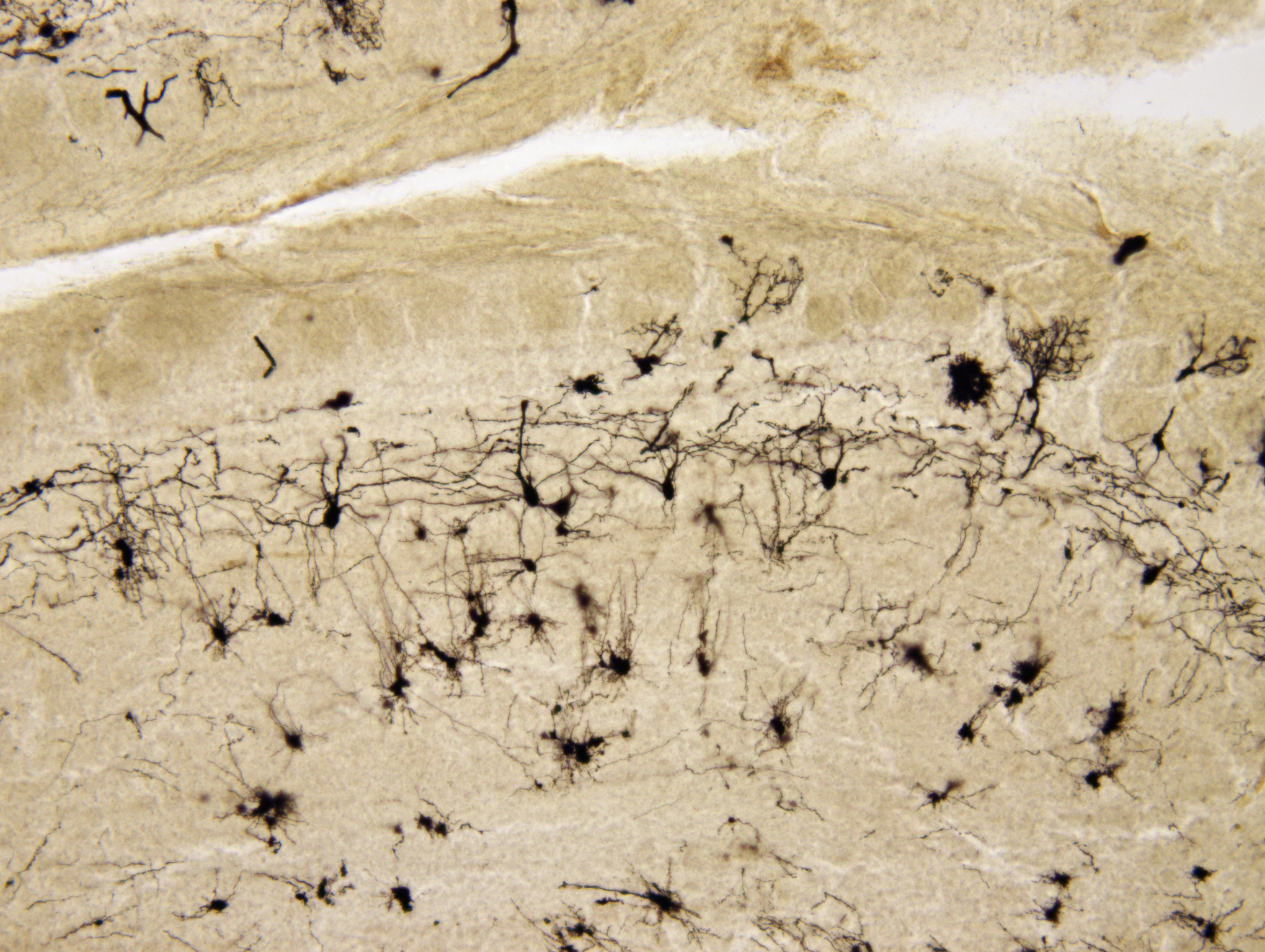

Supplement: Supplementary file 5 — Source data Fig. 3 [file 44319_2024_166_MOESM5_ESM.zip › Source Data for Figure 3/3C/SUM_cko-12d-adult-10x-3.tif]

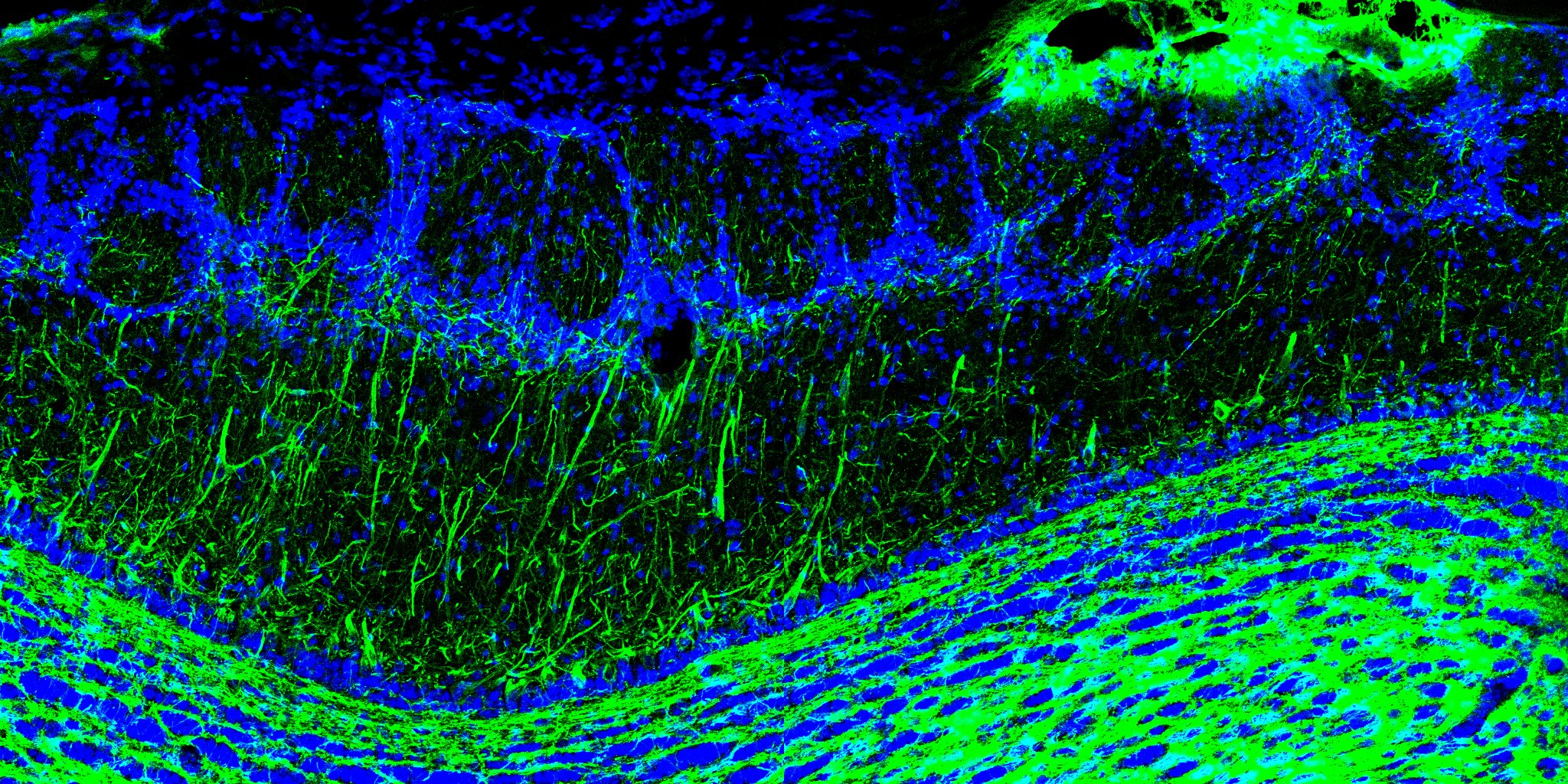

Supplement: Supplementary file 5 — Source data Fig. 3 [file 44319_2024_166_MOESM5_ESM.zip › Source Data for Figure 3/3A/MAX_20200916 WT NF165+DAPI-03.jpg]

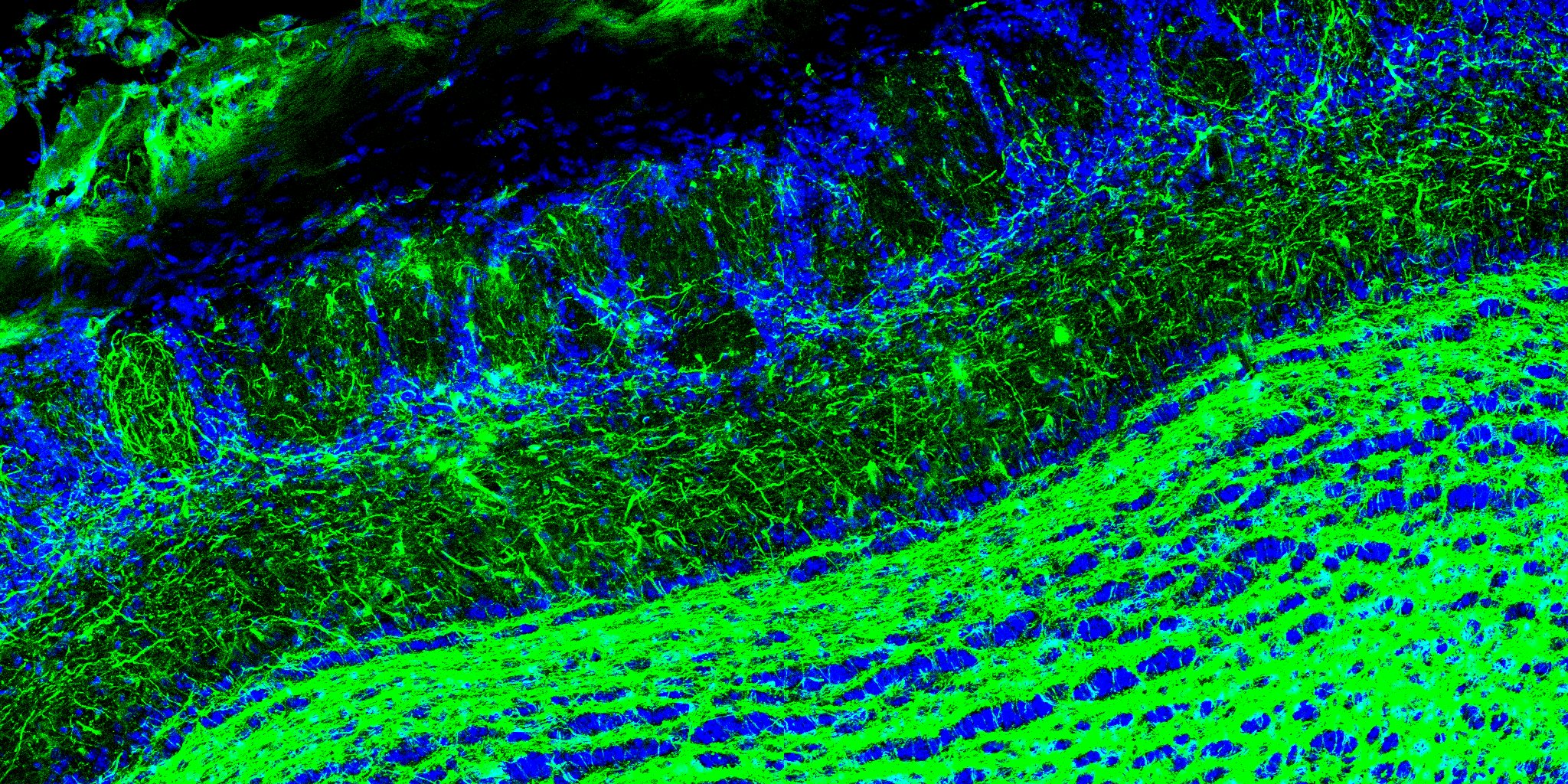

Supplement: Supplementary file 5 — Source data Fig. 3 [file 44319_2024_166_MOESM5_ESM.zip › Source Data for Figure 3/3A/MAX_20200916 KO NF165+DAPI-03.jpg]

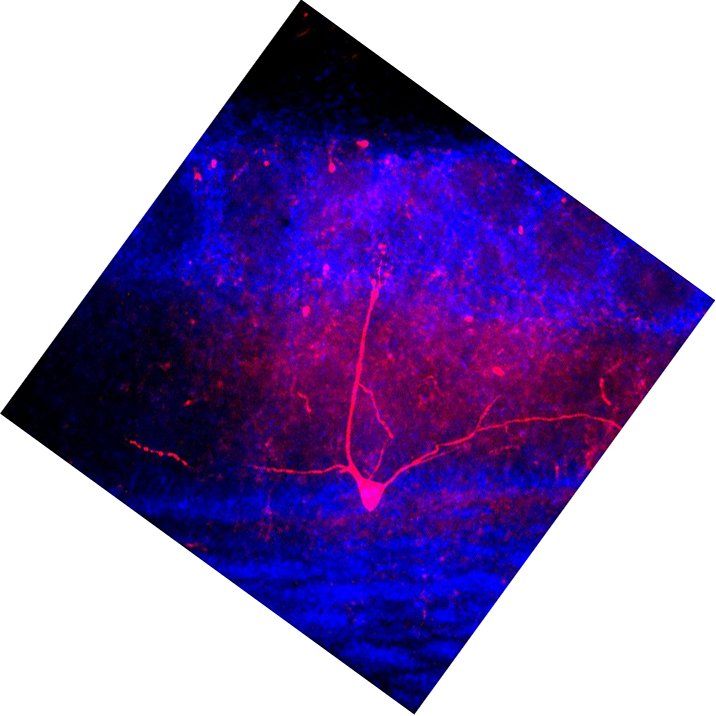

Supplement: Supplementary file 5 — Source data Fig. 3 [file 44319_2024_166_MOESM5_ESM.zip › Source Data for Figure 3/3F/MAX_20230110 WT3A RFP+DAPI-07.jpg]

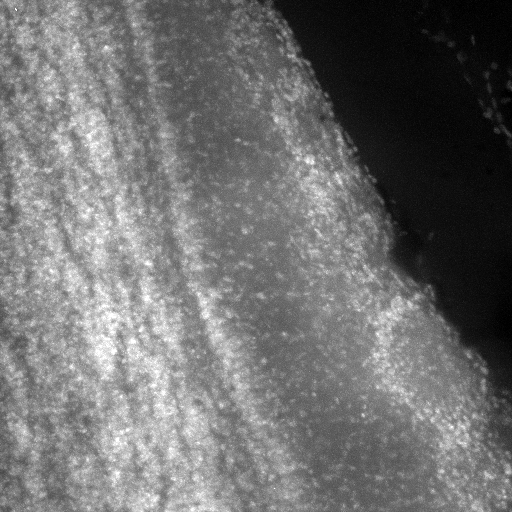

Supplement: Supplementary file 5 — Source data Fig. 3 [file 44319_2024_166_MOESM5_ESM.zip › Source Data for Figure 3/3F/MAX_20230110 C2flox3B RFP+DAPI-02.tif]

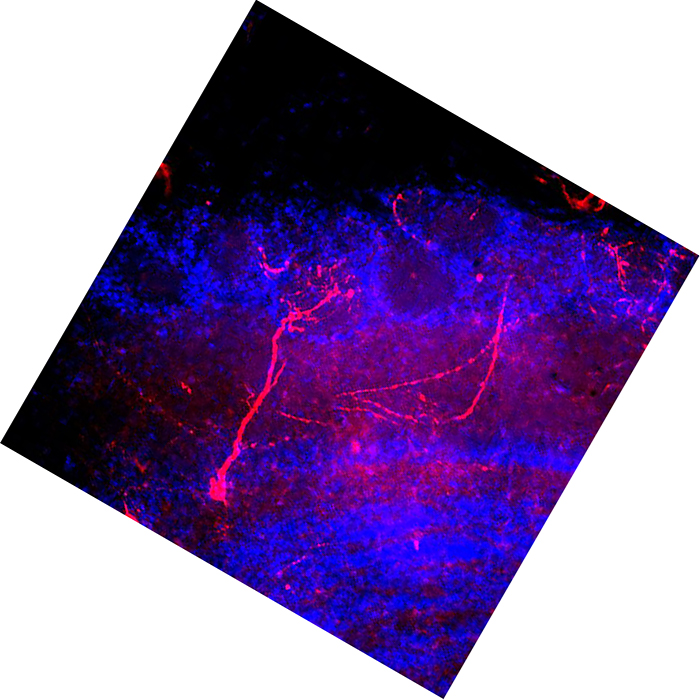

Supplement: Supplementary file 5 — Source data Fig. 3 [file 44319_2024_166_MOESM5_ESM.zip › Source Data for Figure 3/3F/MAX_20230110 WT3A RFP+DAPI-04.jpg]

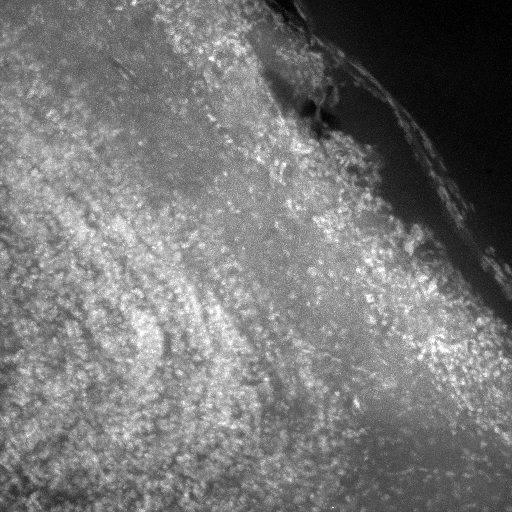

Supplement: Supplementary file 5 — Source data Fig. 3 [file 44319_2024_166_MOESM5_ESM.zip › Source Data for Figure 3/3F/MAX_20230110 C2flox2A RFP+DAPI-24.tif]

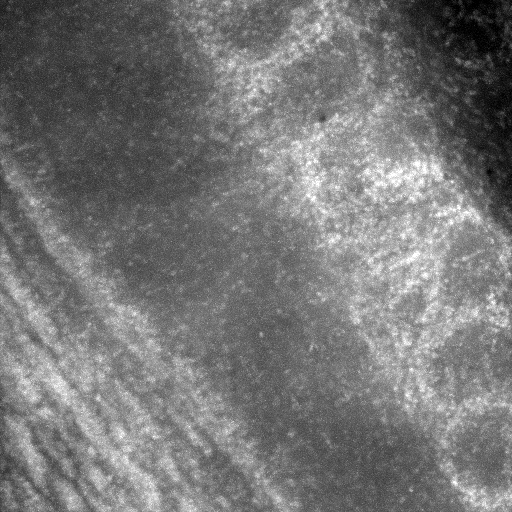

Supplement: Supplementary file 5 — Source data Fig. 3 [file 44319_2024_166_MOESM5_ESM.zip › Source Data for Figure 3/3F/MAX_20230110 WT3A RFP+DAPI-07.tif]

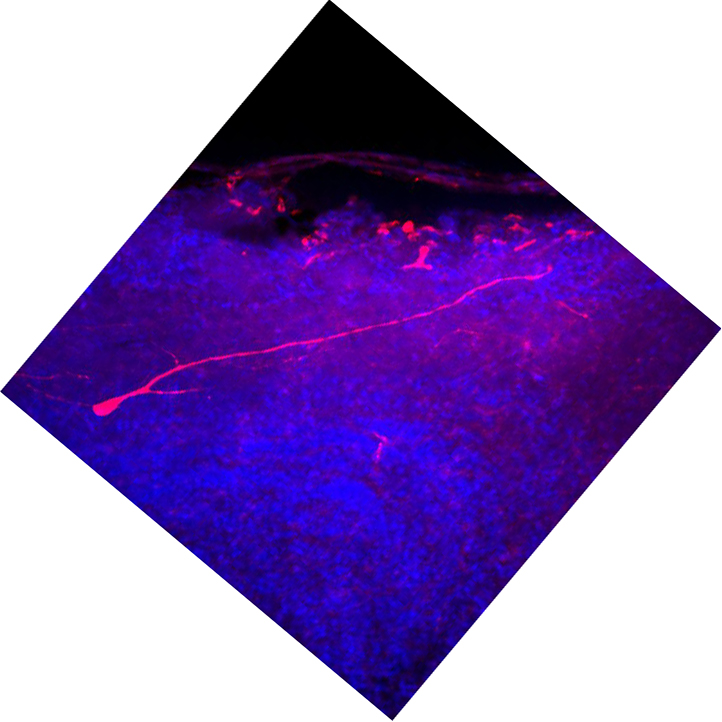

Supplement: Supplementary file 5 — Source data Fig. 3 [file 44319_2024_166_MOESM5_ESM.zip › Source Data for Figure 3/3F/MAX_20230110 C2flox2A RFP+DAPI-24.jpg]

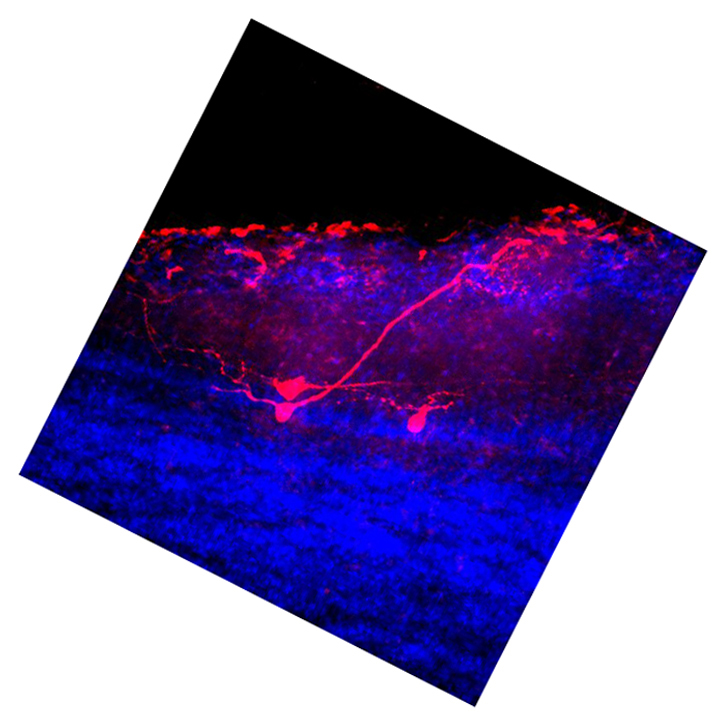

Supplement: Supplementary file 5 — Source data Fig. 3 [file 44319_2024_166_MOESM5_ESM.zip › Source Data for Figure 3/3F/MAX_20230110 C2flox3B RFP+DAPI-02.jpg]

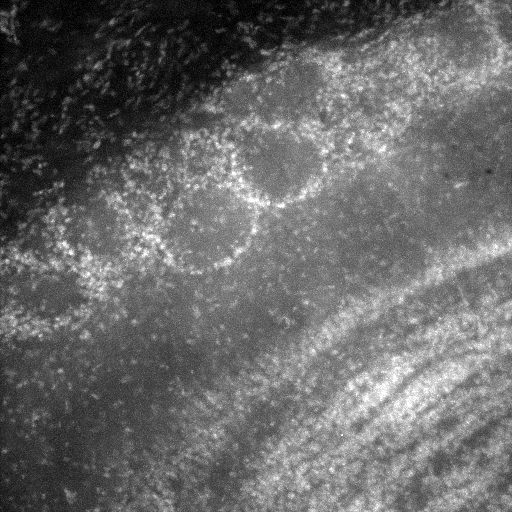

Supplement: Supplementary file 5 — Source data Fig. 3 [file 44319_2024_166_MOESM5_ESM.zip › Source Data for Figure 3/3F/MAX_20230110 WT3A RFP+DAPI-04.tif]

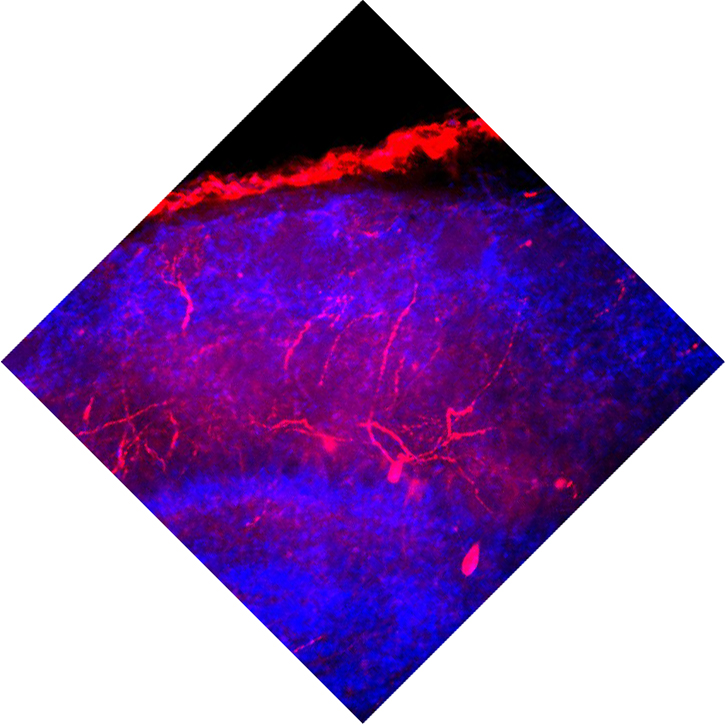

Supplement: Supplementary file 5 — Source data Fig. 3 [file 44319_2024_166_MOESM5_ESM.zip › Source Data for Figure 3/3F/MAX_20230110 C2flox2A RFP+DAPI-11.jpg]

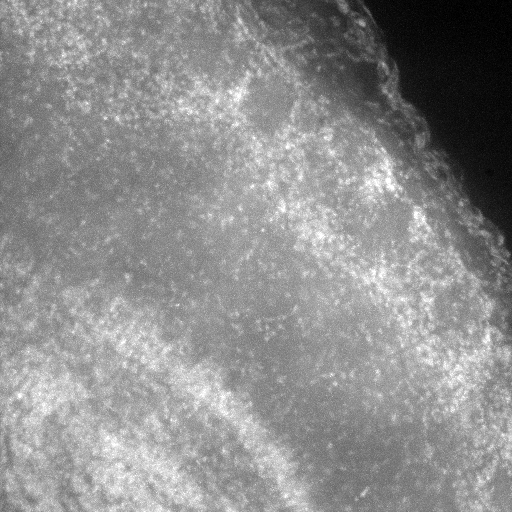

Supplement: Supplementary file 5 — Source data Fig. 3 [file 44319_2024_166_MOESM5_ESM.zip › Source Data for Figure 3/3F/MAX_20230110 C2flox2A RFP+DAPI-11.tif]

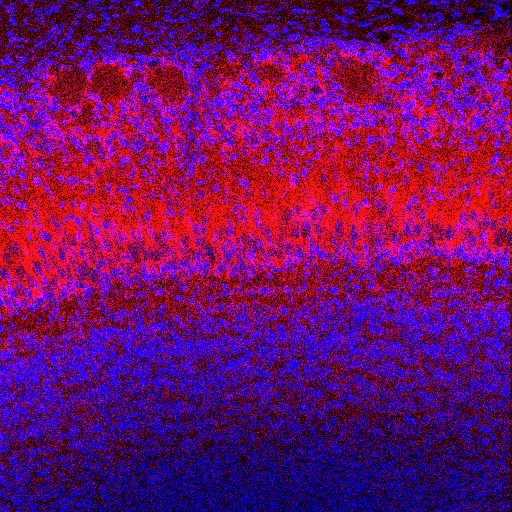

Supplement: Supplementary file 6 — Source data Fig. 4 [file 44319_2024_166_MOESM6_ESM.zip › Source Data for Figure 4/4B/P3 DAPI+CS2.tif]

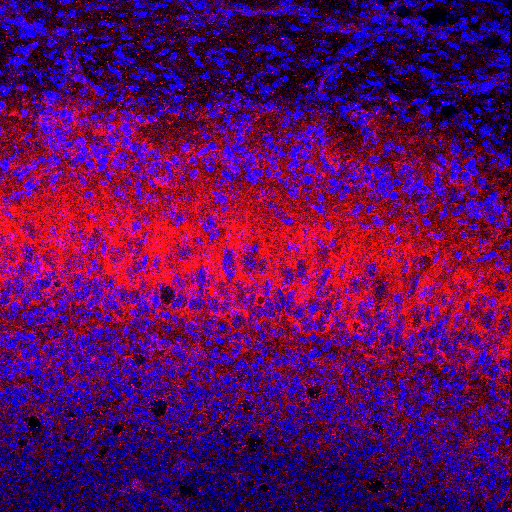

Supplement: Supplementary file 6 — Source data Fig. 4 [file 44319_2024_166_MOESM6_ESM.zip › Source Data for Figure 4/4B/P0 DAPI+CS2.tif]

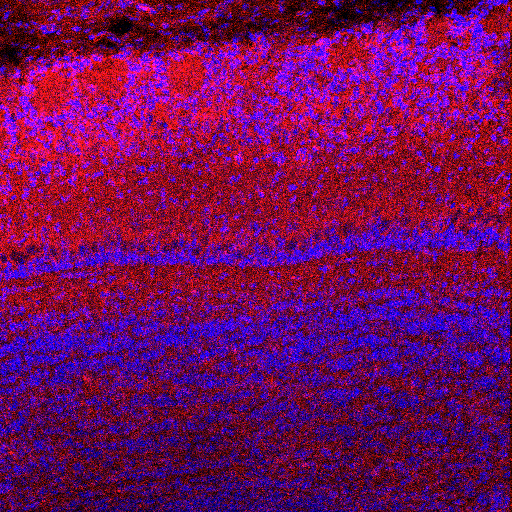

Supplement: Supplementary file 6 — Source data Fig. 4 [file 44319_2024_166_MOESM6_ESM.zip › Source Data for Figure 4/4B/P7 DAPI+CS2.tif]

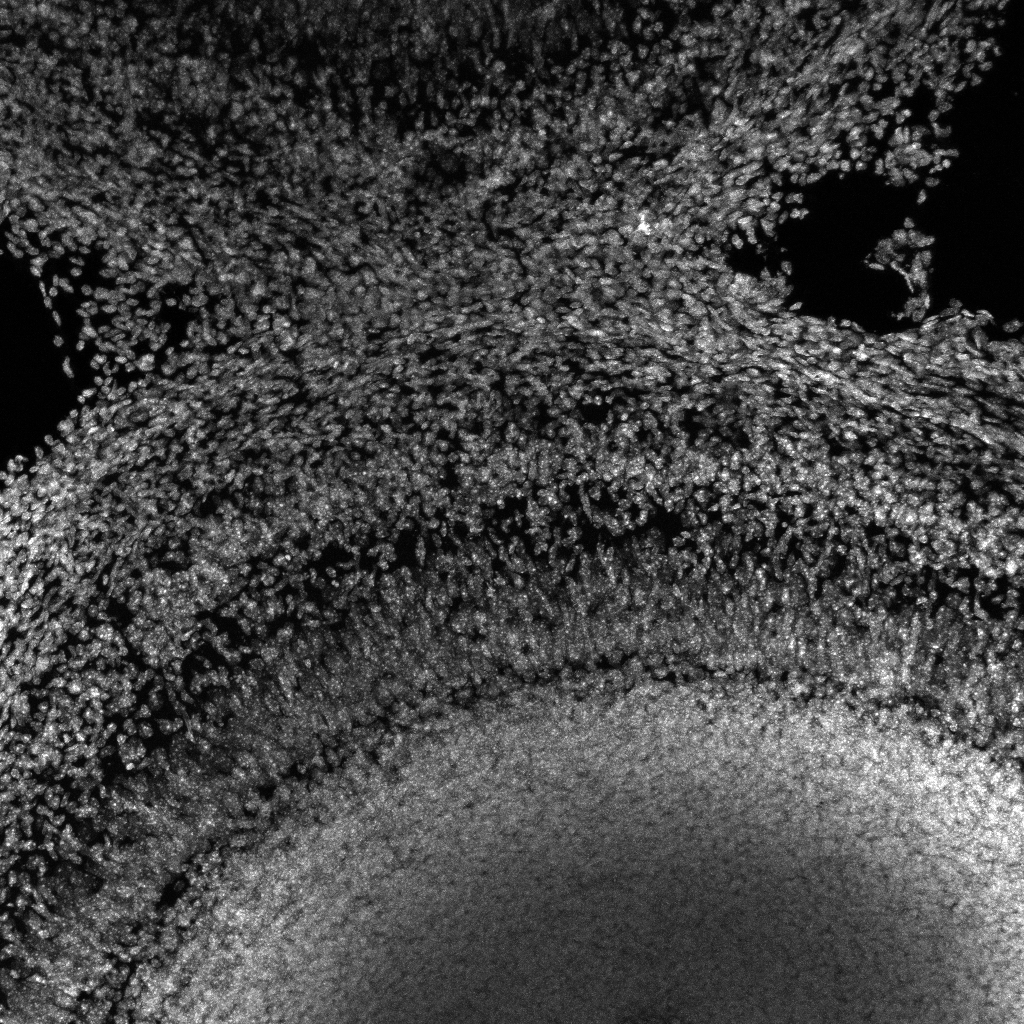

Supplement: Supplementary file 6 — Source data Fig. 4 [file 44319_2024_166_MOESM6_ESM.zip › Source Data for Figure 4/4C/MAX_ WT mScarlet+DAPI-04.tif]

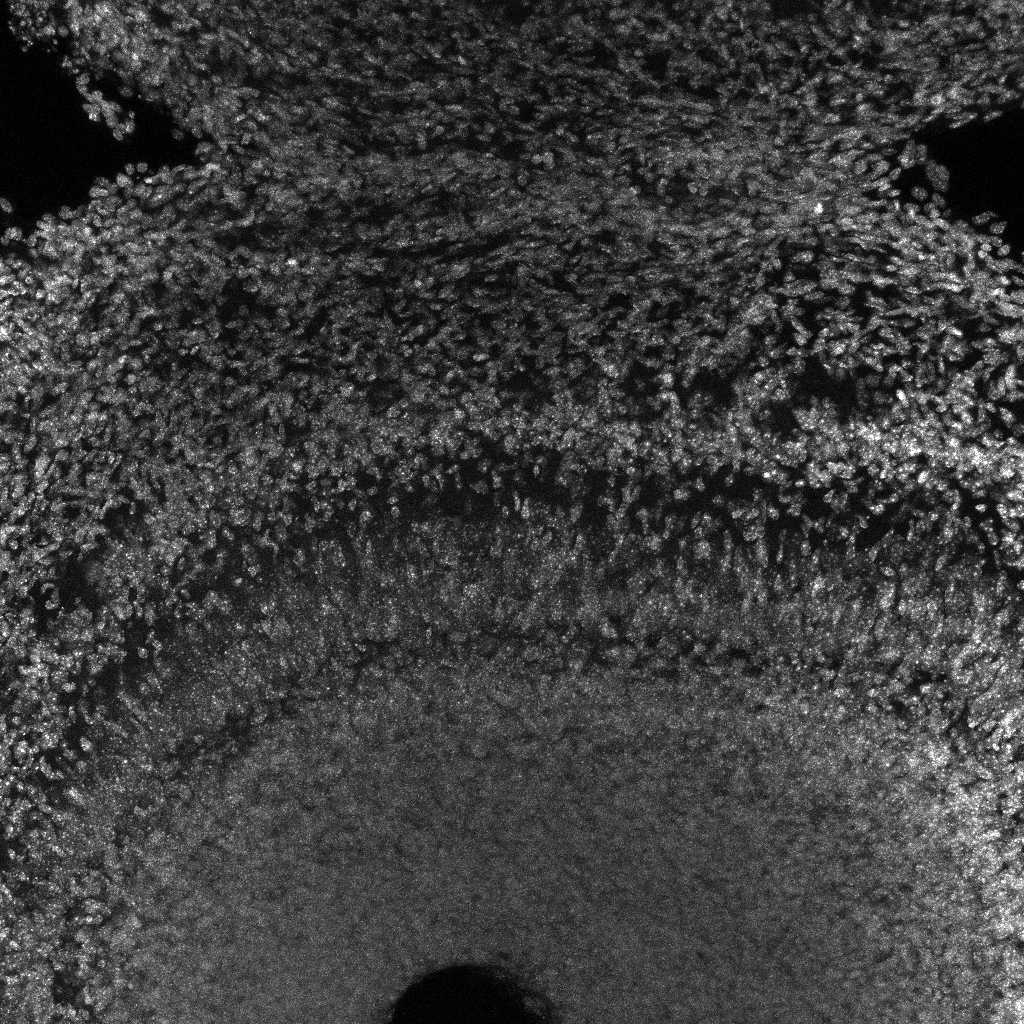

Supplement: Supplementary file 6 — Source data Fig. 4 [file 44319_2024_166_MOESM6_ESM.zip › Source Data for Figure 4/4C/MAX_ P0 C2-flox2 Tbr2+RFP-5Z.tif]

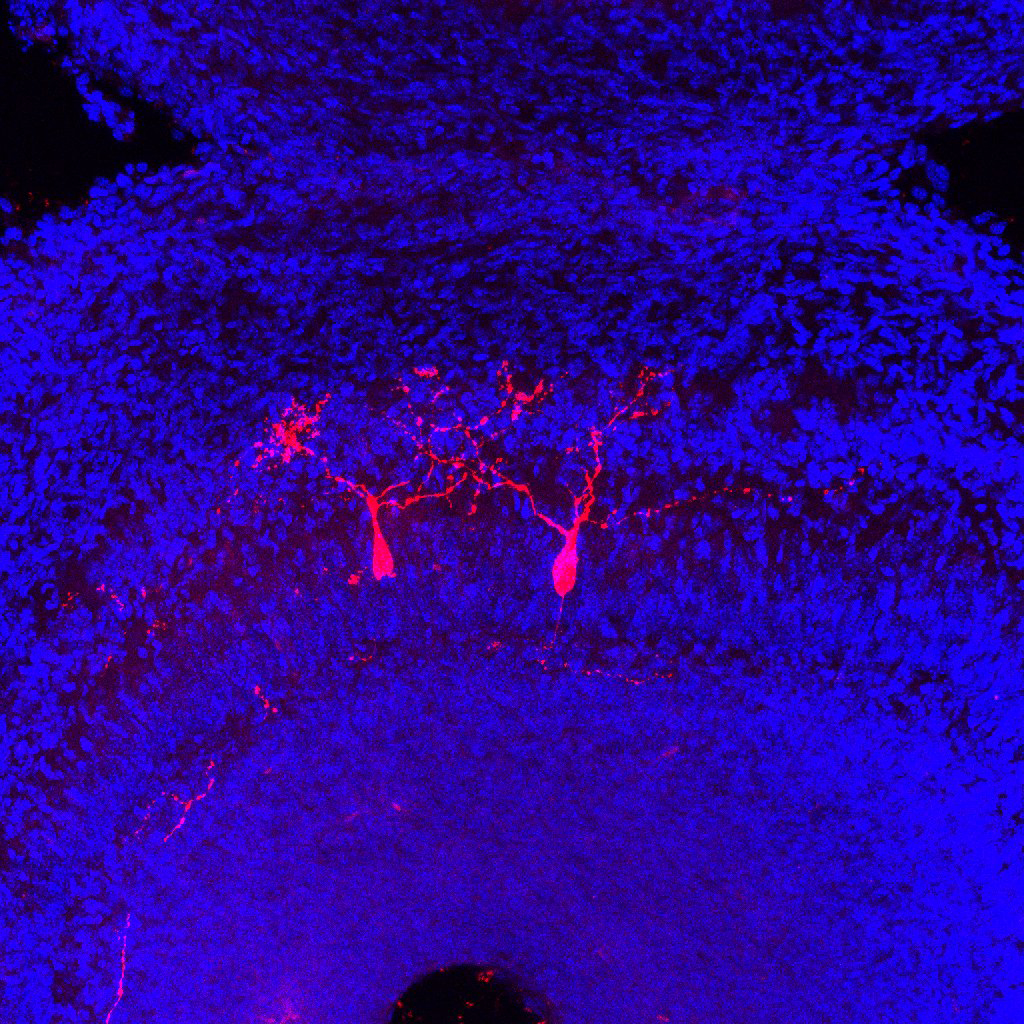

Supplement: Supplementary file 6 — Source data Fig. 4 [file 44319_2024_166_MOESM6_ESM.zip › Source Data for Figure 4/4C/MAX_ P0 C2-flox2 Tbr2+RFP-5Z.jpg]

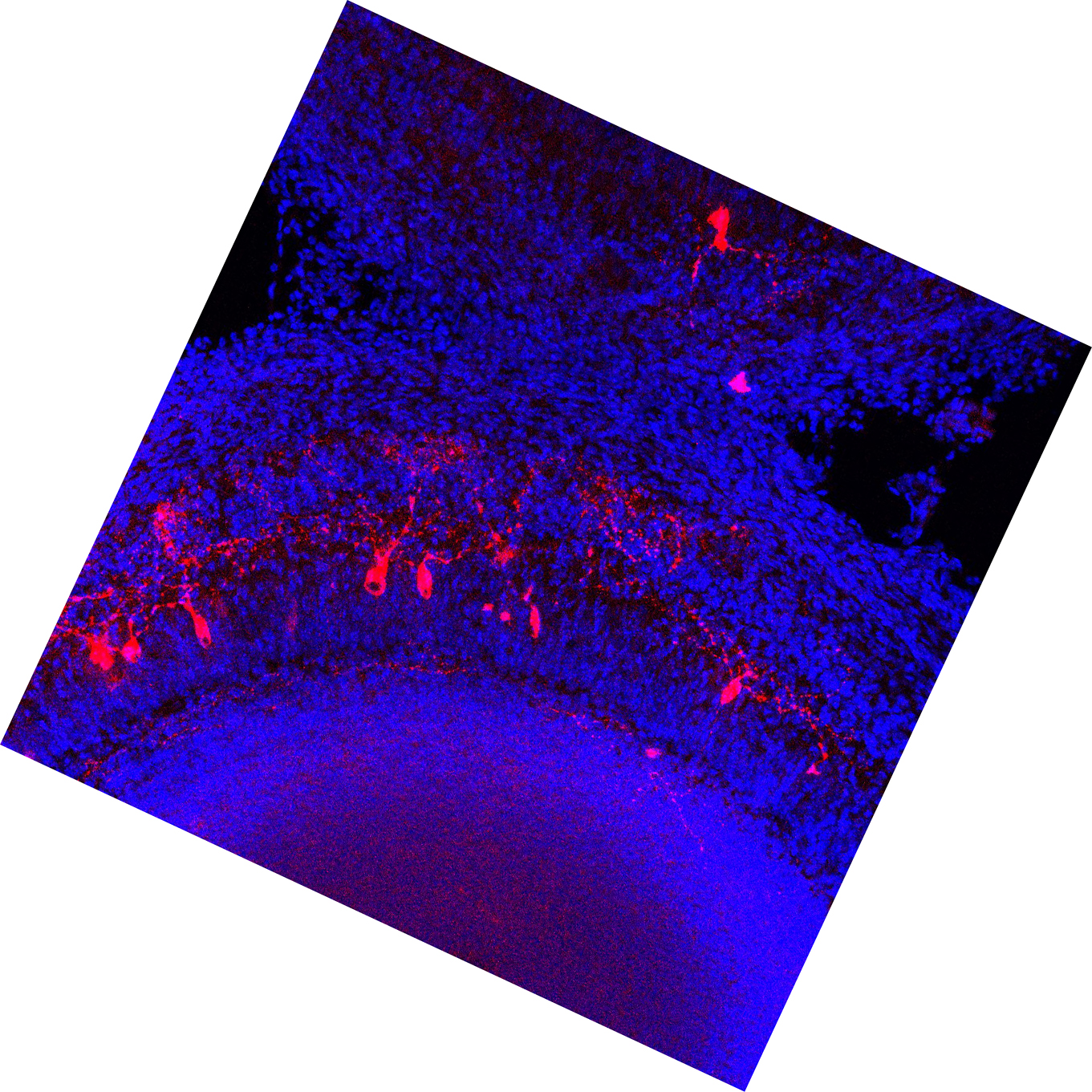

Supplement: Supplementary file 6 — Source data Fig. 4 [file 44319_2024_166_MOESM6_ESM.zip › Source Data for Figure 4/4C/MAX_ WT mScarlet+DAPI-04.jpg]

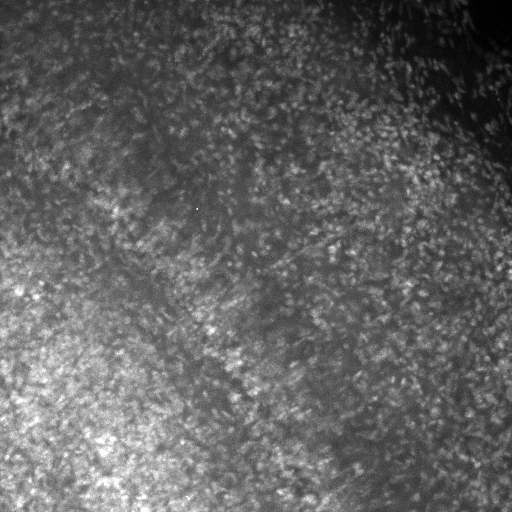

Supplement: Supplementary file 6 — Source data Fig. 4 [file 44319_2024_166_MOESM6_ESM.zip › Source Data for Figure 4/4D/MAX_F3 RFP+DAPI-09.tif]

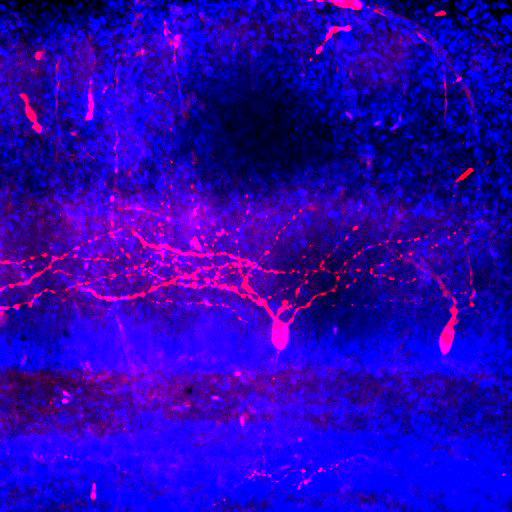

Supplement: Supplementary file 6 — Source data Fig. 4 [file 44319_2024_166_MOESM6_ESM.zip › Source Data for Figure 4/4D/MAX_C2-flox 3 DAPI+RFP-08.jpg]

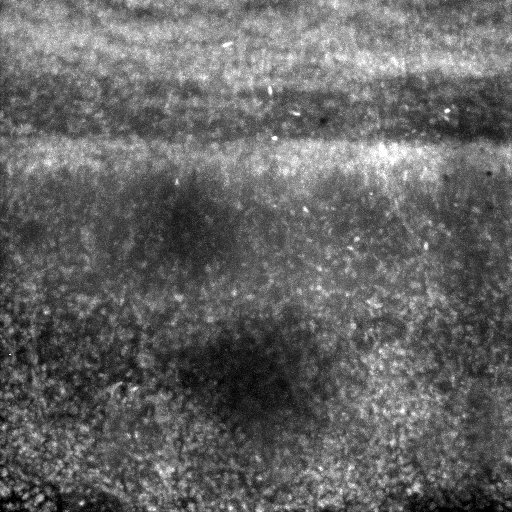

Supplement: Supplementary file 6 — Source data Fig. 4 [file 44319_2024_166_MOESM6_ESM.zip › Source Data for Figure 4/4D/MAX_C2-flox 3 DAPI+RFP-08.tif]

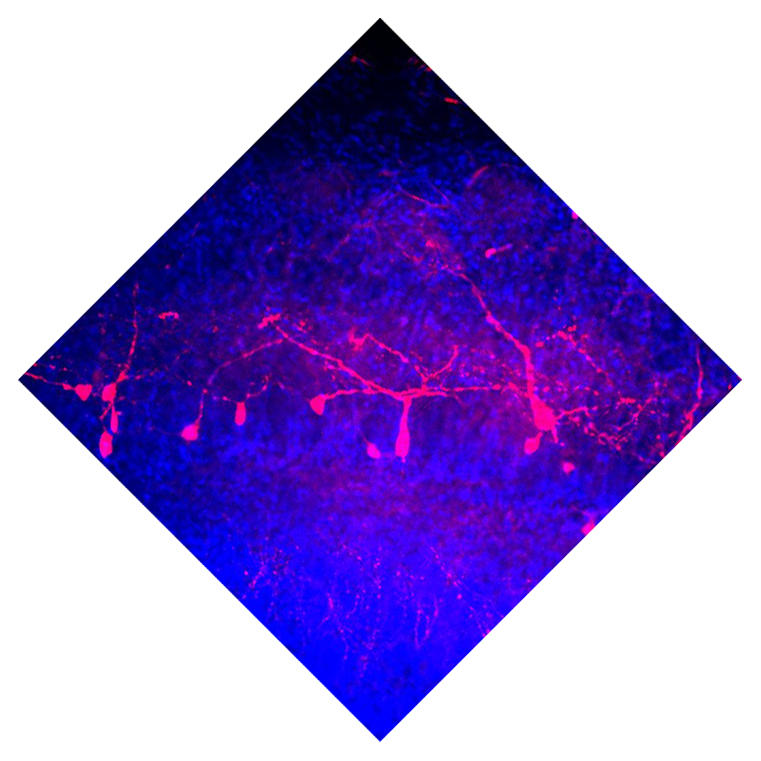

Supplement: Supplementary file 6 — Source data Fig. 4 [file 44319_2024_166_MOESM6_ESM.zip › Source Data for Figure 4/4D/MAX_F3 RFP+DAPI-09.jpg]

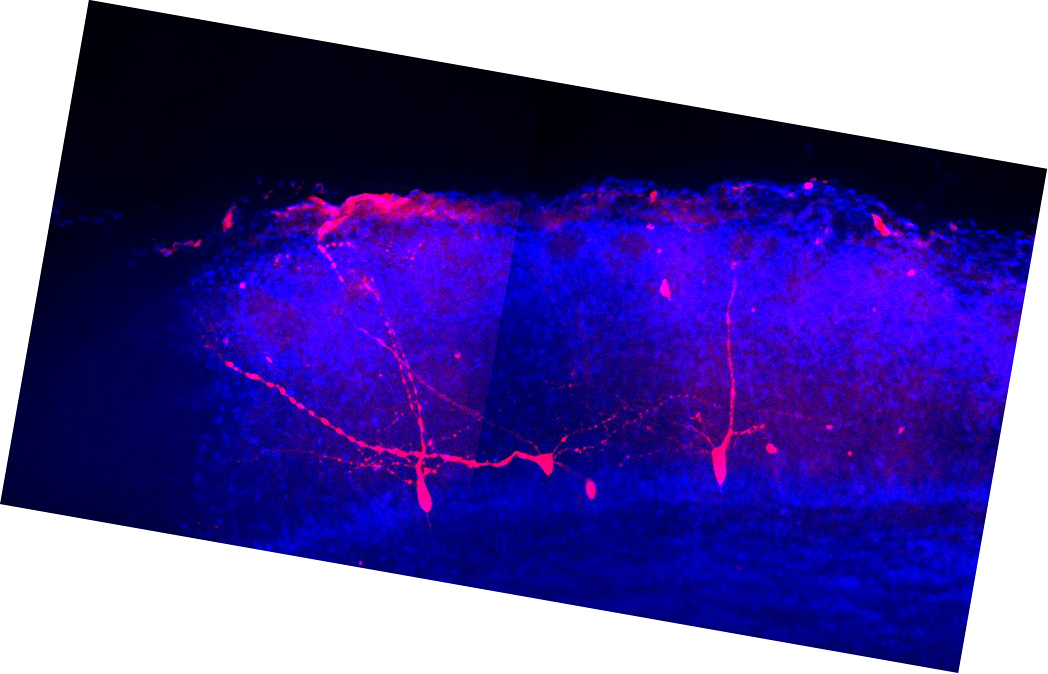

Supplement: Supplementary file 6 — Source data Fig. 4 [file 44319_2024_166_MOESM6_ESM.zip › Source Data for Figure 4/4D/MAX_WT1 DAPI+RFP-04.czi #1-1.jpg]

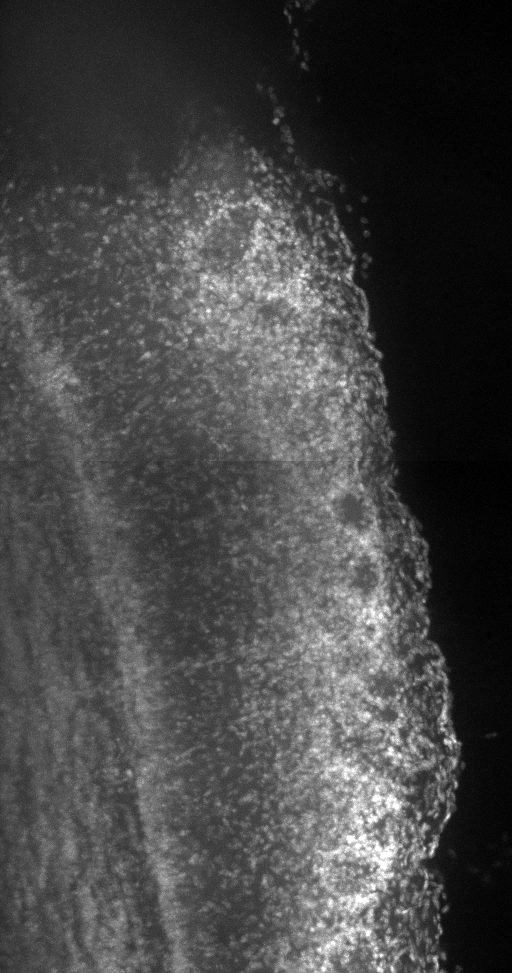

Supplement: Supplementary file 6 — Source data Fig. 4 [file 44319_2024_166_MOESM6_ESM.zip › Source Data for Figure 4/4D/MAX_WT1 DAPI+RFP-04.czi #1-1.tif]
