# Supplementary material for: Deficiency of CAMSAP2 impairs olfaction and the morphogenesis of mitral cells
Source: EMBO Rep. 2024 Jun 5;25(7):7. doi: 10.1038/s44319-024-00166-x (PMC11239855; doi:10.1038/s44319-024-00166-x)
Supplement: Supplementary file 7 — Expanded View Figures [file 44319_2024_166_MOESM7_ESM.pdf]

Expanded View Figures

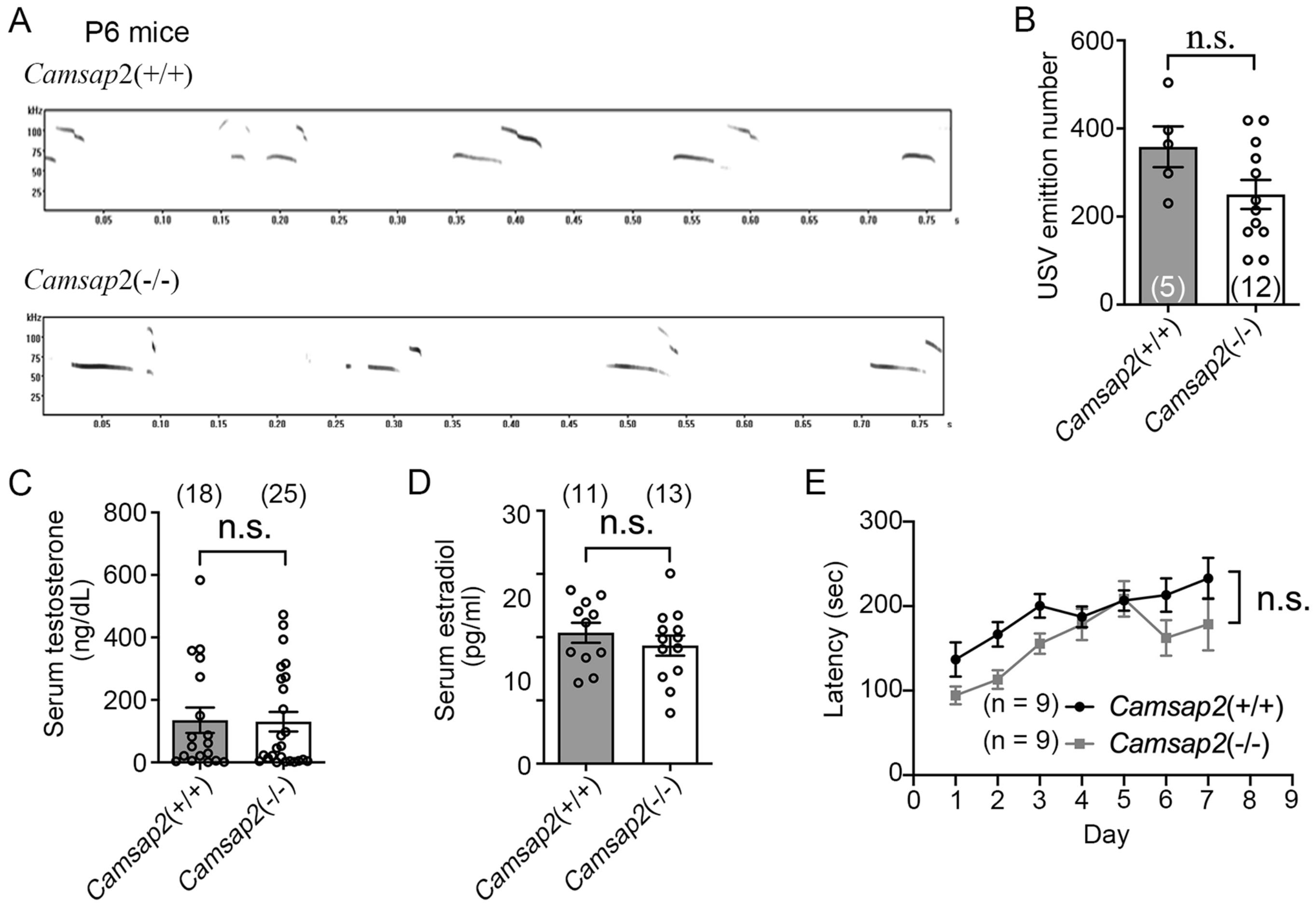

Figure EV1. Hormone levels and motor function are not responsible for the infertility of *Camsap2*(−/−) male mice.

(**A**) Representative spectrograms of USVs emitted by P6 mice. (**B**) Quantification of the number of USVs emitted by P6 mice (unpaired Student's *t* test, $n = 5$ or 12 biological replicates). (**C**) The concentration of serum testosterone (unpaired Student's *t* test, $n = 18$ or 25 biological replicates). (**D**) The concentration of serum estradiol (unpaired Student's *t* test, $n = 11$ or 13 biological replicates). (**E**) Motor performance on an accelerating rotarod, no significant difference exists at any time point (two-way R-M ANOVA, $n = 9$ biological replicates). Data information: Data are represented as mean ± SEM. n.s. not significant, $P > 0.05$.

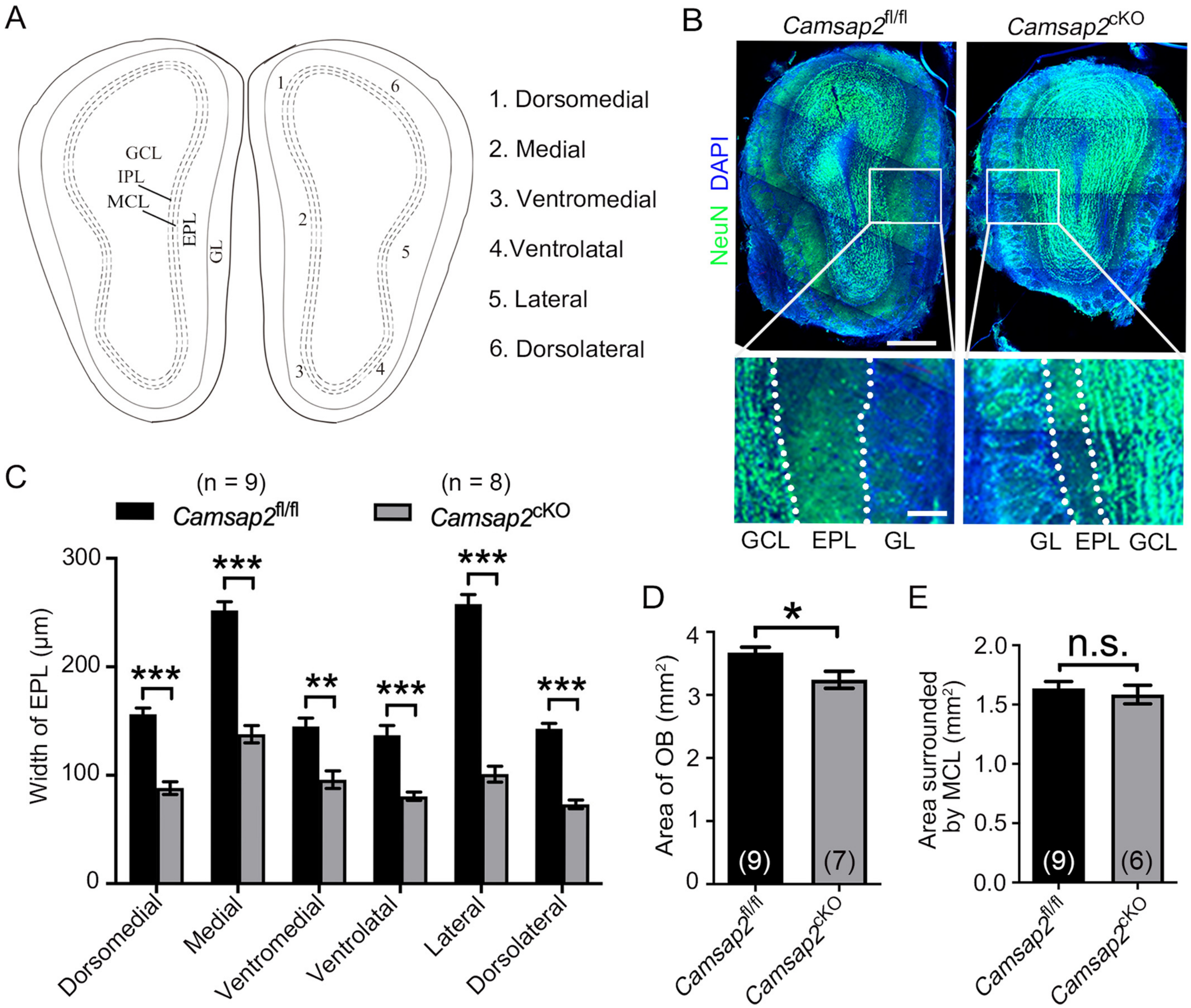

Figure EV2. Knockout of *Camsap2* diminishes the thickness of EPL in the OB.

(**A**) Schematic diagram of the OB. (**B**) Representative images of the OB from *Camsap2*$^{fl/fl}$ and *Camsap2*^{cKO} mice, mature neurons were immunostained with antibody anti-NeuN. (**C**) Quantification of the thickness of EPL from *Camsap2*$^{fl/fl}$ and *Camsap2*^{cKO} mice (two-way R-M ANOVA with Šídák's multiple comparisons test, $n = 3$ biological replicates, ***$P = 0.0009$ or < 0.0001, **$P = 0.0033$). (**D**) Quantification of the area of the OB from *Camsap2*$^{fl/fl}$ and *Camsap2*^{cKO} mice (unpaired Student's *t* test, $n = 3$ biological replicates, *$P = 0.0221$). (**E**) Quantification of the area surrounded by MCL from *Camsap2*$^{fl/fl}$ and *Camsap2*^{cKO} mice (unpaired. Student's *t* test, $n = 3$ biological replicates). Data information: Data are represented as mean ± SEM. n.s. not significant, $P > 0.05$. Scale bars: 400 μm and 100 μm in full-size images and zoomed areas.

© The Author(s)

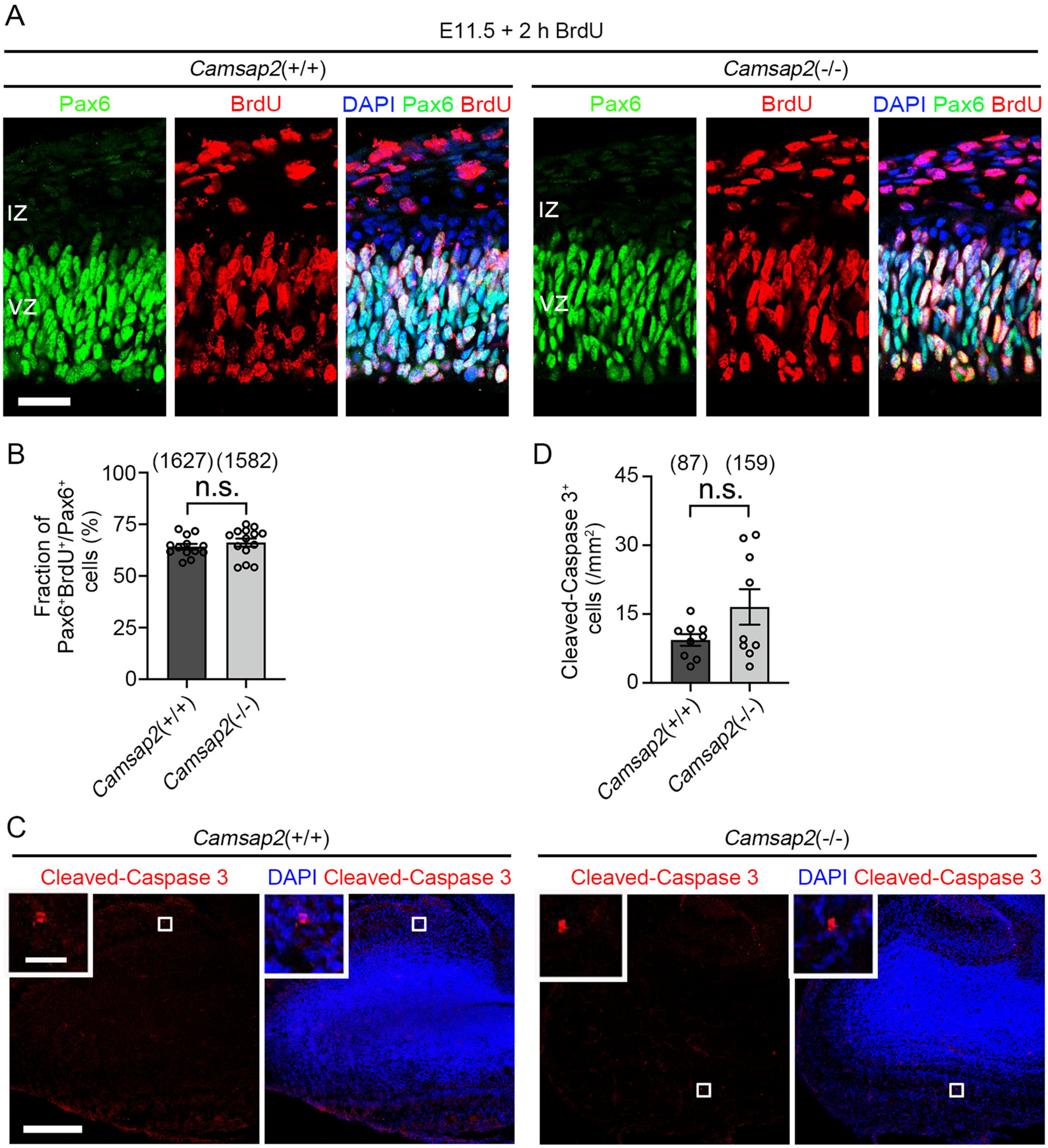

Figure EV3. CAMSAP2 is dispensable for the neurogenesis and apoptosis of MCs.

(**A**) Representative images of coronal OB sections from E11.5 mice. The progenitor cells and proliferating. cells are immunostained with antibodies against Pax6 and BrdU after BrdU labeling for 2 h. (**B**) The percentage of proliferating progenitor cells (unpaired Student's *t* test, $n = 4$ biological replicates). (**C**) Representative images of sagittal OB sections from P0 mice. The cells undergoing apoptosis are immunostained with antibody against Cleaved-Caspase 3 (Activated-Caspase 3). (**D**) The density of Cleaved-Caspase 3^+ cells in OB (unpaired Student's *t* test, $n = 3$ biological replicates). Data information: Data are represented as mean ± SEM. n.s. not significant, $P > 0.05$. Scale bars: (**A**), 30 μm; (**C**) 300 μm and 30 μm in full-size images and zoomed areas.

© The Author(s)

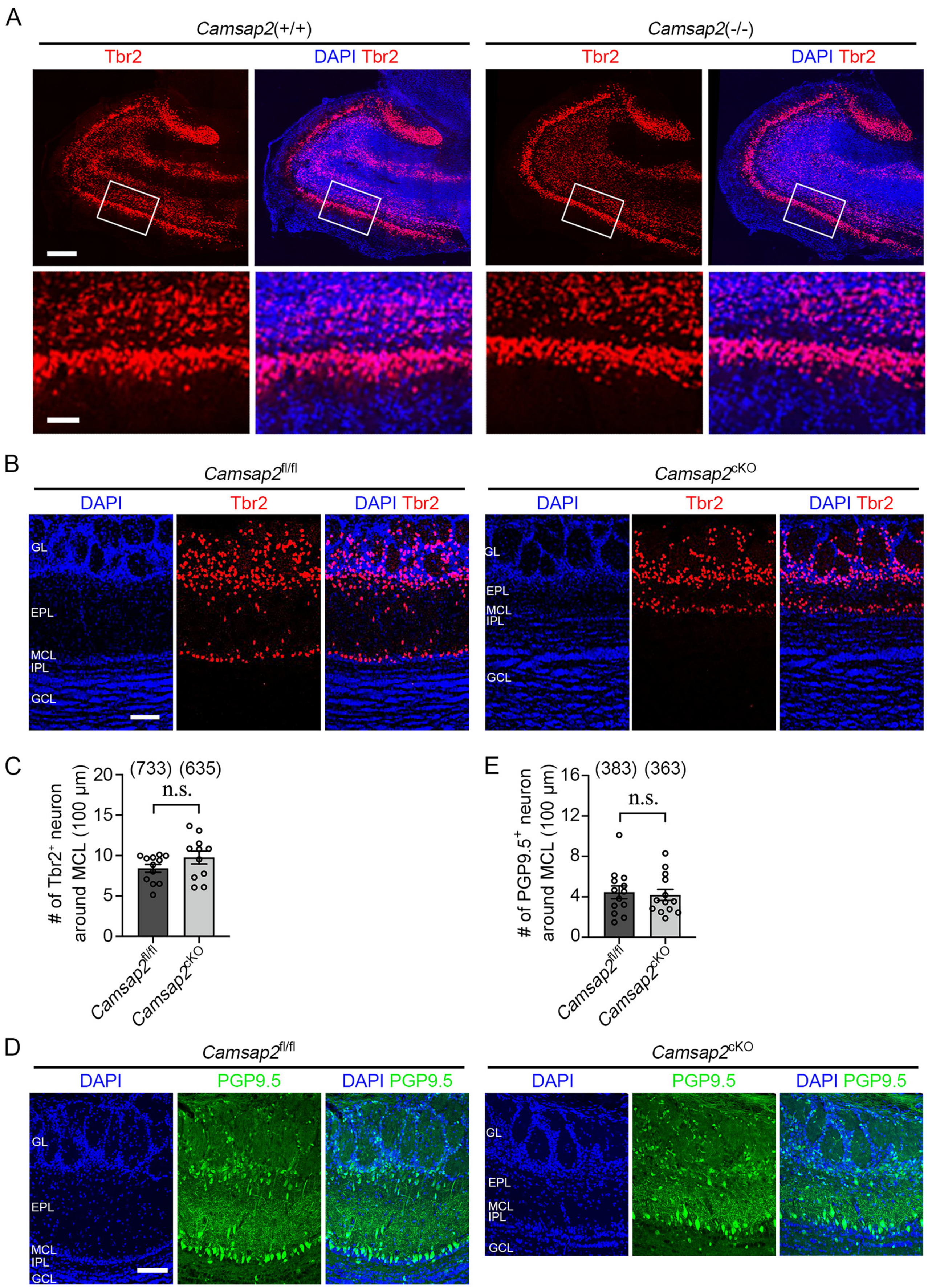
A
Camsap2(+/+)
Camsap2(-/-)
Tbr2
DAPI Tbr2
B
Camsap2fl/fl
Camsap2cKO
DAPI
Tbr2
DAPI Tbr2
GL
EPL
MCL
IPL
GCL
C
# of Tbr2+ neuron around MCL (100 μm)
(733) (635)
n.s.
20
15
10
5
0
Camsap2fl/fl
Camsap2cKO
E
# of PGP9.5+ neuron around MCL (100 μm)
(383) (363)
n.s.
16
12
8
4
0
D
DAPI
PGP9.5
DAPI PGP9.5

 © The Author(s)

Figure EV4. CAMSAP2 is dispensable for the migration of MCs.

(**A**) Representative images of the sagittal sections of the OB from P0 mice. The MCs are immunostained with antibody against Tbr2. (**B**) Representative images of coronal sections of the OB of adult mice. The MCs are immunostained with. antibody against Tbr2. (**C**) Quantification of the number of $Tbr2^{+}$ neurons around the MCL (unpaired Student's *t* test, $n = 3$ biological replicates). (**D**) Representative images of coronal sections of the OB of adult mice. The MCs are immunostained with. antibody against PGP9.5. (**E**) Quantification of the number of PGP9.5 positive neurons around the MCL (unpaired Student's *t* test, $n = 3$ biological replicates). Data information: Data are represented as mean ± SEM. n.s. not significant, $P > 0.05$. Scale bars: (**A**) 200 μm and 50 μm in full-size images and zoomed areas; (**B**, **D**) 400 μm.

© The Author(s)
